# Complex Investigation of the Similarities and Differences between Ten Commercially Available Human Serum Albumin Preparations

**DOI:** 10.1021/acsomega.5c06541

**Published:** 2025-09-25

**Authors:** Rita Jakabfi-Csepregi, Zoltán Nagymihály, Zoltán Horváth-Szalai, Balázs Szirmay, Éva Varga-Visi, Omeralfaroug Ali, Edward Agyarko, András Szabó, Miklós Poór

**Affiliations:** 1 Department of Laboratory Medicine, Medical School, 37656 University of Pécs, Ifjúság útja 13, Pécs H-7624, Hungary; 2 Molecular Medicine Research Group, János Szentágothai Research Centre, 37656 University of Pécs, Ifjúság útja 20, Pécs H-7624, Hungary; 3 Agribiotechnology and Precision Breeding for Food Security National Laboratory, Institute of Physiology and Nutrition, Department of Physiology and Animal Health, 72406 Hungarian University of Agriculture and Life Sciences, Guba Sándor u. 40, Kaposvár H-7400, Hungary; 4 HUN-REN-MATE Mycotoxins in the Food Chain Research Group, 72406 Hungarian University of Agriculture and Life Sciences, Guba Sándor u. 40, Kaposvár H-7400, Hungary

## Abstract

Human serum albumin (HSA) maintains the oncotic pressure in the blood, also having buffer and antioxidant functions. Furthermore, numerous ligand molecules are circulating dominantly in albumin-bound form in the intravascular water space. The formation of stable ligand–albumin complexes commonly has significant physiological, pharmacological, and/or toxicological importance. Regarding ligand–HSA interactions, certain studies show controversial results that may have partly resulted from the differences between the protein preparations applied. To test this hypothesis, 10 HSA preparations were obtained, and then their albumin and free fatty acid content, fructosamine and free thiol levels, antioxidant capacity, fluorescence emission spectra, and ligand binding ability were examined. Our results demonstrate that sometimes even major differences can be observed when we compare these proteins. Therefore, the precise specification and the careful selection of HSA preparations seem to be reasonable.

## Graphical abstract

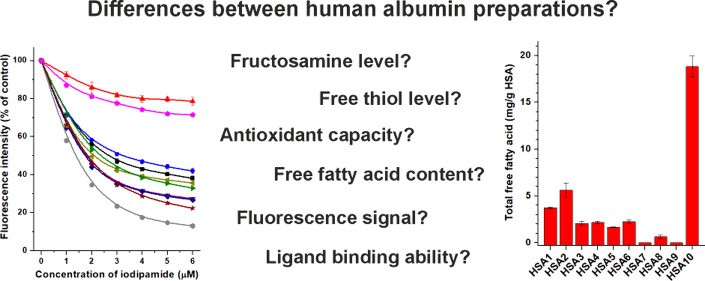

## 1 Introduction

Human serum albumin (HSA) is a major plasma protein with high concentrations (35–50 mg/mL ≈ 500–750 μM) and significant ligand binding capacity in the circulation.^1^ HSA can interact with several drugs, xenobiotics, nutrients, and endogenous compounds in the blood; the formation of highly stable ligand–albumin complexes can strongly affect the pharmacokinetics/toxicokinetics of certain molecules.^2,3^ Furthermore, HSA can be used as a drug carrier to optimize drug delivery^4,5^ and it is also applied in the treatment of hypoalbuminemia and sepsis.^6,7^ There are nine fatty acid binding sites (FA1-FA9) on HSA.^1^ The two main drug binding sites are Sudlow’s Site I (FA7) and Sudlow’s Site II (FA3–FA4).^1,2^ Site I is a hydrophobic cavity in subdomain IIA; its typical ligands are warfarin, furosemide, phenylbutazone, and iodipamide.^2^ Site II is a large cavity in subdomain IIIA, which is occupied, for example, by diazepam, ibuprofen, ketoprofen, and naproxen.^2^ Another important binding site involved in the albumin binding of certain drugs is the Heme pocket (FA1).^8^ The heme site is located in a cavity of subdomain IB, its typical ligands are hemin, bilirubin, and biliverdin.^1,8^

In the scientific literature, we frequently find large differences in the binding constants of ligand–HSA complexes (e.g., for flavonoids^9,10^ or for hemin^8,11,12^ ). Several explanations can be assumed, including the improper experimental design and/or data evaluation, no correction of background signals and/or the inner-filter effect, etc.^13,14^ Nevertheless, another possible reason can be the differences in the quality/purity of HSA preparations used. Previous studies demonstrated that albumin preparations can sometimes differ in posttranslational modifications, redox state, antioxidant properties, and accompanying proteins.^15–17^ In a recent study, the interactions of eight ligands (warfarin, azapropazone, diflunisal, indomethacin, lidocaine, thyroxine, diclofenac, and diazepam) were examined with five HSA preparations applying rapid equilibrium dialysis: most of the proteins represented similar binding abilities, while some of them showed relatively large differences in their interactions with warfarin (site I), diazepam (site II), or lidocaine (heme site).^18^ In addition, major discrepancies were observed regarding the immobilization yields of certain HSA preparations on magnetic nanoparticles.^18^

Only few earlier studies evaluated and compared the properties/interactions of commercially available HSA preparations (see details in the previous paragraph). We assumed that some of the HSA preparations may show considerable differences, which can affect their ligand binding ability. To test the similarities/differences between the commercially available HSA preparations, we purchased 10 products (lyophilized powder form), where fatty acid free, globulin free, and recombinant preparations were also included (Table 1). Thereafter, the following parameters were examined: HSA content (based on bromocresol green colorimetric method), electrophoresis, fructosamine level, free thiol concentration, antioxidant capacity, free fatty acid content, fluorescence emission spectra, impacts of site markers (site I, site II, and heme site) on the emission signals of the proteins, and interactions with site I and site II ligands based on ultrafiltration experiments. Our results demonstrate that some HSA preparations can show considerable discrepancies in the above-listed properties/interactions. The complex experimentation applied gives a deeper insight into the potential differences between HSA preparations and draws the attention that we cannot consider these proteins as completely equivalent.

**Table 1:** Major Identification Codes and Properties of HSA Preparations Examined in the Current Study

abbreviation	producer	product code	lot number	purity	other properties
HSA1	Merck (Sigma-Aldrich)	A1653	SLCD1119	≥96%	
HSA2	Merck (Sigma-Aldrich)	A9511	SLBS5934	≥97%	
HSA3	Merck (Sigma-Aldrich)	126658	3892542	≥95%	
HSA4	Merck (EMD Millipore)	12666	3856206	≥95%	low heavy metals (≤20 ppm)
HSA5	Merck (EMD Millipore)	126654	3870449	≥95%	
HSA6	Merck (EMD Millipore)	SRP6516	8A24L75460	≥95%	
HSA7	Merck (Sigma-Aldrich)	A1887	SLCB2530	≥96%	essential fatty acid free (≤0.007% FA)
HSA8	Merck (Sigma-Aldrich)	A8763	SLCD9951	≥99%	essential globulin free
HSA9	Merck (Sigma-Aldrich)	A3782	SLCM4058	≥99%	fatty acid free (≤0.1% FA) and globulin free
HSA10	Merck (Sigma-Aldrich)	A9731	SLBL9344V	≥96%	recombinant (expressed in rice)

## 2 Materials and Methods

### 2.1 Materials

Human serum albumin (HSA; see detailed descriptions in Table 1), furosemide (F4381), iodipamide (I0639), diazepam (D0899), ketoprofen (K1751), biliverdin hydrochloride (30891), warfarin (45706), naproxen (PHR1040), luminol (3-aminophthalhydrazide; A8511), 4-iodophenol (I1020-1), Trolox (6-hydroxy-2,5,7,8-tetramethylchroman-2-carboxylic acid; 238813), horseradish peroxidase (POD; 77332), hydrogen peroxide (H_2_O_2_; 19990), and ammonium persulfate (A3678) were purchased from Merck (Darmstadt, Germany). Methyl orange (9572) was from Reanal (Budapest, Hungary). Glycerol (40057), sodium dodecyl sulfate (SDS; 17B024103), Tris (tris­(hydroxymethyl)­aminomethane; 103156X), and glacial acetic acid (20099.290) were obtained from VWR (Debrecen, Hungary). Beta-mercaptoethanol (1610710), tetramethylethylenediamine (TEMED; 1610800), and Coomassie Brilliant blue R 250 (CBB; 42660) were from Bio-Rad Laboratories (Hercules, California, USA). Acrylamide and *N*,*N*-methylene-bis-acrylamide (A4983) were purchased from Thermo Fisher Scientific (Waltham, Massachusetts, USA).

### 2.2 Quantification of HSA Content

To compare HSA content, 40 mg/mL (≈600 μM) solutions were prepared from each lyophilized powder in PBS, and then the bromocresol green colorimetric method was carried out applying the Cobas 8000/c502 module (05166861190; Roche Diagnostics, Mannheim, Germany) at our accredited clinical laboratory (Department of Laboratory Medicine, Medical School, University of Pécs, Hungary; NAH-9-0008/2021).

### 2.3 Electrophoresis

For SDS-PAGE (sodium dodecyl sulfate polyacrylamide gel electrophoresis), human albumin stock solutions (4 mg/mL, dissolved in PBS) were further diluted with 5-fold concentrated Laemmli sample buffer (50% glycerol, 25% beta-mercaptoethanol, and 10% SDS in 0.3125 M Tris–HCl buffer (pH 6.8)) to 2 mg/mL albumin concentration. Thereafter, samples were vortexed and heated in a water bath for 3 min at 100 °C. A 10 μL volume of each sample was loaded onto the wells of the stacking gel. In a single gel, five different HSA solutions and the molecular weight marker (Precision Plus Protein All Blue Standard, 161-0373; Bio-Rad Laboratories, Hercules, California, USA) were loaded. Denaturing discontinuous one-dimensional 10% SDS-PAGE^19^ was performed with Mini-Protean Dual Vertical Electrophoresis Cell (Bio-Rad). Gel slabs for electrophoresis were manually prepared by using Mini-Protean Glass Plates. After electrophoresis, gels were placed into CBB staining solution to visualize individual protein bands.^20^ Further details of the electrophoresis and staining are described in the Supporting Information. CBB-stained gels were documented with a camera on a Kaiser Slimlite light box.

### 2.4 Determination of Fructosamine Levels

Fructosamine levels were measured with a colorimetric assay on the Cobas 8000/c702 module (05171962190; Roche) at our accredited clinical laboratory (Department of Laboratory Medicine, Medical School, University of Pécs, Hungary; NAH-9-0008/2021).

### 2.5 Measurement of Free Thiol Groups

Ellman’s reagent (5,5′-dithio-bis­(2-nitrobenzoic acid); 22582; Thermo Fisher Scientific, Waltham, Massachusetts, USA) was used to quantify the free thiol groups according to the manufacturer’s instructions. Briefly, Ellman’s reagent (4 mg/mL) was prepared in the reaction buffer (0.1 M sodium phosphate, pH 8.0, with 1 mM EDTA). A 250 μL volume of samples (40 mg/mL HSA, dissolved in PBS) or standards (cysteine, 0.0–1.5 mM) was mixed with 50 μL of Ellman’s reagent and 2.5 mL of reaction buffer and then incubated for 15 min at room temperature. The absorbance was measured at 412 nm using a PerkinElmer EnSpire Multimode plate reader (Waltham, Massachusetts, USA). Thiol content was calculated using a cysteine standard curve.

### 2.6 Testing the Antioxidant Effects

The total antioxidant capacity (TAC) was assessed using a luminol-based enhanced chemiluminescence (ECL) microplate assay, as it has been earlier described.^21^ Briefly, a 20 μL of blank, standard, or sample (40 mg/mL HSA, dissolved in PBS) was pipetted into the wells of white 96-well microplates (6005290; Per-Form Hungaria Ltd., Budapest, Hungary). Subsequently, 270 μL of the POD-ECL solution^21^ was added and the plate was shaken for 10 s. ECL reaction was initiated by the automated injection of 20 μL of H_2_O_2_ solution (1.5 mM). The luminescence of the reaction was monitored for 10 min (with 64 s measurement intervals), applying a Biotek Synergy HT plate reader (Agilent, Santa Clara, California, USA). TAC values were calculated based on the total light output (area under the curve, AUC) of the standards (Trolox, 0–100 μM). TAC of the samples was determined as Trolox equivalent (TE) in μM using a calibration curve.

### 2.7 Determination of Free Fatty Acid Content

HSA samples were processed exactly according to Logsdon et al.^22^ Then, samples were analyzed with a Shimadzu 2030 NEXIS GC FID equipment (Shimadzu, Kyoto, Japan). The injected volume was 3 μL, and the split ratio was 1:10. Separation was performed on a Zebron ZB-WAXPLUS capillary column (30 m × 0.25 mm × 0.25 μm film, Phenomenex, Torrance, California, USA). Operating conditions included an injector temperature of 220 °C, a detector temperature of 250 °C, and a helium flow rate of 28 cm/s. The oven temperature was programmed as follows: starting from 60 °C with a 2 min hold, increasing to 150 °C at a rate of 13 °C/min, and then from 150 to 180 °C at a rate of 2 °C/min with a 10 min hold at 180 °C, and finally from 180 to 220 °C at a rate of 2 °C/min with a 14 min hold at 220 °C (total duration: 72 min).

The chromatographic evaluation was performed with the LabSolutions 5.93 software, using the PostRun module (Shimadzu, Kyoto, Japan) with manual peak integration. The identification of the fatty acids was performed based on the retention time values of a CRM external standard (Supelco 37 Component FAME Mix, CRM47885, Merck).

### 2.8 Fluorescence Spectroscopic Studies

Fluorescence measurements were performed with a Hitachi F-4500 fluorescence spectrophotometer (Tokyo, Japan) at room temperature. Emission spectra of HSA solutions were recorded using 295 nm excitation wavelength. In quenching studies, increasing levels (final concentrations: 0, 1, 2, 3, 4, 5, and 6 μM) of site I (furosemide and iodipamide), site II (diazepam and ketoprofen), or heme site (biliverdin and methyl orange) markers were added to albumin (2 μM), and then their quenching effects were evaluated at 340 nm. After background correction, the inner-filter effects of ligands were also corrected based on the following equation:^23^

$$I_{cor}=I_{obs}\times 10^{\left(A_{ex}+A_{em}\right)/2}$$

where *I*_cor_ and *I*_obs_ are the corrected and observed fluorescence emission signals at 340 nm, respectively. Furthermore, *A*_ex_ and *A*_em_ are the absorbance values of the site markers applied at 295 and 340 nm, respectively. Stern–Volmer quenching constants (*K*_SV_) were determined with linear fitting, employing the following equation:^24^

$$\frac{I_{0}}{I}=1+K_{SV}\times \left[Q\right]$$

where *I*_0_ marks the fluorescence emission signal of HSA without the ligand, *I* denotes the emission intensity of HSA in the presence of the ligand, and [*Q*] is the molar concentration of the quencher.

### 2.9 Ultrafiltration Studies

In ultrafiltration experiments, we applied our previously reported models.^25,26^ Briefly, Amicon Ultra centrifugal filters (30 kDa; Merck, Darmstadt, Germany) were washed once with water and once with PBS (each 500 μL). Then, samples (500 μL) were filtered for 10 min (centrifugation: 7500*g*, 25 °C, fixed angle rotor). Since HSA and albumin-bound molecules cannot pass through the filter with a 30 kDa cutoff value, HSA retains the bound ligand molecules, resulting in the decreased concentrations of site markers in the filtrate.^25,26^ In the first experiment, the site I marker warfarin (1.0 μM) was filtered without and with HSA (3.5 or 6.0 μM). In the second experiment, the site II marker naproxen (1.0 μM) was filtered in the absence and presence of albumin (1.0 or 1.6 μM). After ultrafiltration, the concentrations of warfarin and naproxen were quantified in the filtrates with the previously described HPLC-FLD and HPLC-UV methods, respectively.^25,27^

### 2.10 Statistical Analyses

Figures and tables represent means ± standard deviation (SD) values from three independent experiments. Statistical analyses were carried out with SPSS Statistics software (version 24.0; IBM, Armonk, New York, USA) using one-way ANOVA and Tukey’s post hoc tests. Values of *p* < 0.01 were considered as statistically significant.

## 3 Results

### 3.1 Albumin Content and Electrophoresis of HSA Preparations

After HSA solutions (each 40 mg/mL) were prepared in PBS, albumin levels were quantified based on a colorimetric assay. Most of the HSA preparations did not show statistically significant differences (Table S1), while this assay showed approximately 10% lower albumin levels of HSA10 compared to the other proteins (*p* < 0.01; Figure 1A). In each preparation, SDS-PAGE demonstrated dominant bands between the 50 and 75 kDa molecular weight markers, representing their high HSA (66.5 kDa) content (Figure 1B).

**Figure 1:**
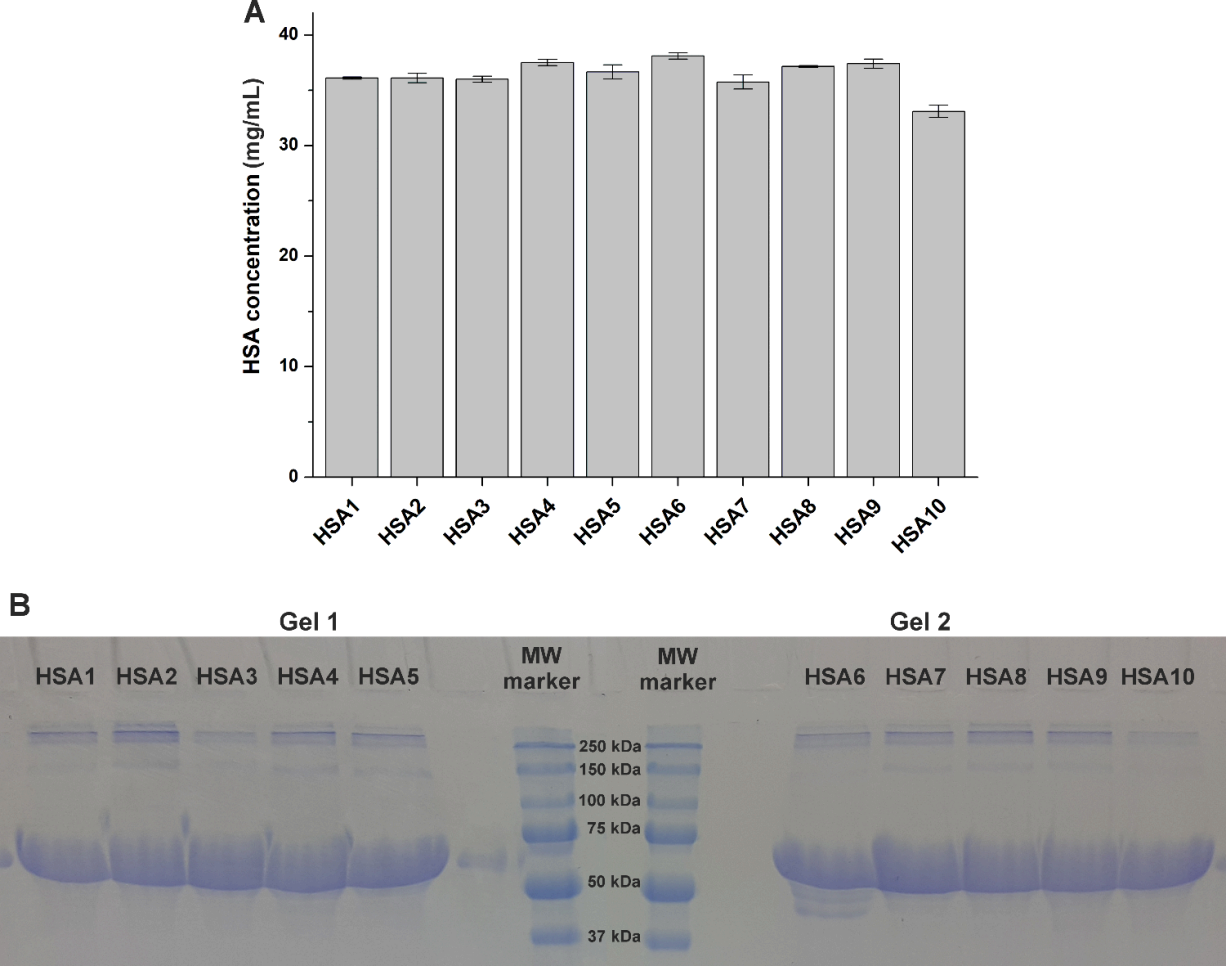
Albumin levels of HSA solutions were determined based on bromocresol green colorimetric method (A; see statistical evaluation in Table S1), where 40 mg of the proteins was dissolved in 1 mL of PBS. SDS-PAGE analysis of the HSA preparations examined (B).

### 3.2 Fructosamine Levels

In the next step, the fructosamine levels of HSA solutions (40 mg/mL) were quantified, where we noticed major differences between the products examined (Figure 2). The highest fructosamine concentration was noticed in HSA2 (284 μM), followed by HSA10 and HSA9 (around 180 μM), and then HSA7, HSA1, and HSA8 (95–120 μM), and finally HSA3–6 (45–60 μM). Thus, an approximately sixfold difference can be noticed between the highest and the lowest levels determined.

**Figure 2:**
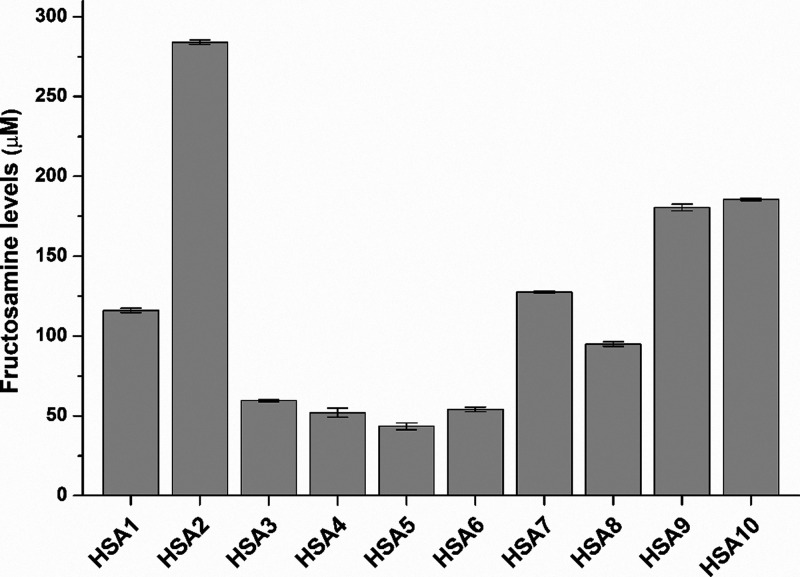
Fructosamine levels of HSA solutions (40 mg/mL in PBS; see statistical evaluation in Table S2).

### 3.3 Free Thiol Groups

The highest level of free thiol groups was observed in HSA1 (173 μM), followed by HSA7–10 (around 140 μM) and then HSA2, HSA3, and HSA6 (between 80 and 100 μM), finally HSA4 and HSA5 (approximately 70 μM and 55 μM, respectively). These data demonstrate major variations in the free thiol content of the HSA preparations examined (Figure 3), where even threefold differences can occur.

**Figure 3:**
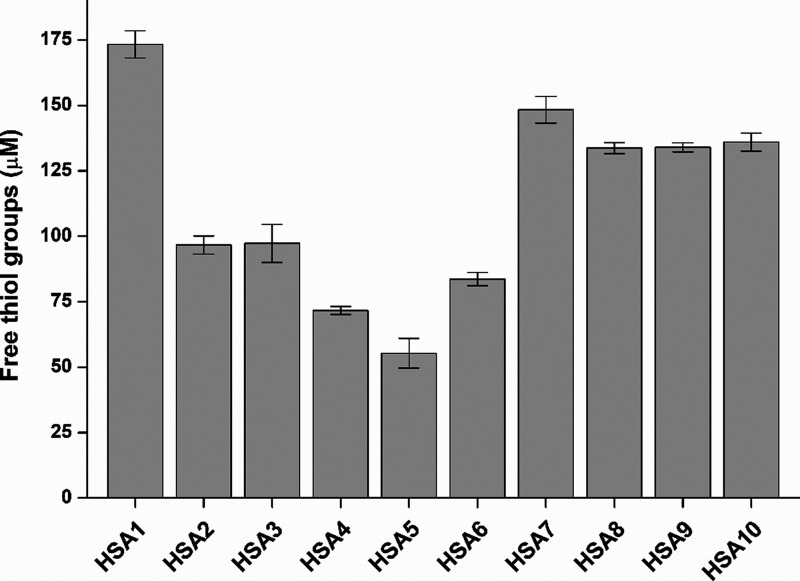
Free thiol groups in HSA solutions (40 mg/mL in PBS; see statistical evaluation in Table S3).

### 3.4 Antioxidant Effects

The antioxidant effects of HSA1–9 were relatively similar. HSA3 and HSA8 showed statistically significant (*p* < 0.01) but only slightly lower antioxidant capacity compared to albumin preparations like HSA1, HSA7, and HSA10 (Figure 4). However, the impact of HSA10 proved to be the highest, distinguishing it from the other preparations.

**Figure 4:**
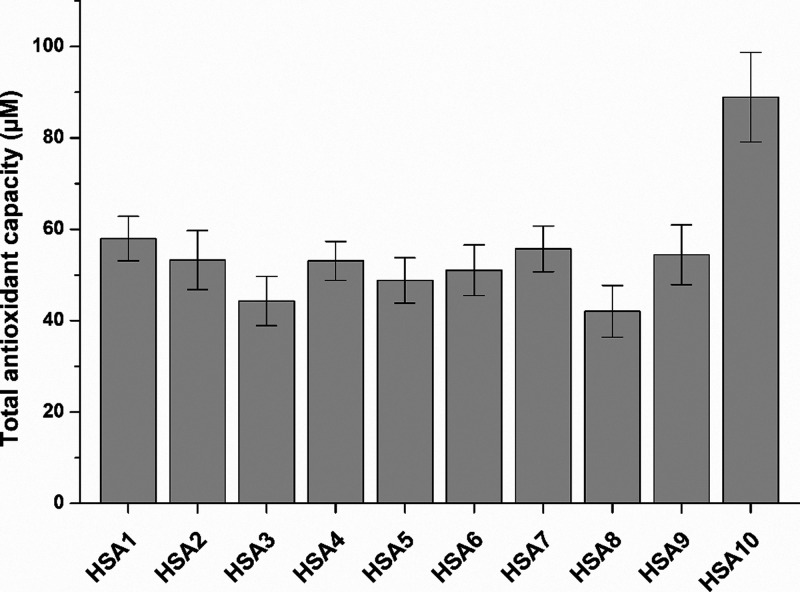
Total antioxidant capacity (in Trolox equivalent) of HSA solutions (40 mg/mL in PBS; see statistical evaluation in Table S4).

### 3.5 Fatty Acid Content

The levels of lauric acid, myristic acid, palmitic acid, palmitoleic acid, stearic acid, oleic acid, and linoleic acid were quantified in the HSA preparations. Among these fatty acids, lauric acid (0–53 μg/g HSA), myristic acid (0–208 μg/g HSA), palmitoleic acid (0–145 μg/g HSA), and stearic acid (0–295 μg/g HSA) were found only at low concentrations (Figure S1). The highest levels of these fatty acids were in the following proteins: lauric acid in HSA2, myristic acid in HSA10, palmitoleic acid in HSA2, and stearic acid in HSA1. Furthermore, the dominant fatty acids detected were palmitic acid (0.0–2.8 mg/g HSA), oleic acid (0.0–2.0 mg/g HSA), and linoleic acid (0.0–13.8 mg/g HSA) (Figure 5). HSA10 contained the highest levels of these three fatty acids. The sum of the seven fatty acid levels is also demonstrated as “total fatty acid”. HSA7 and HSA9 (sold as fatty acid free proteins; Table 1) contained no or negligible amounts, and the total fatty acid level in HSA8 (0.6 mg/g HSA) was also very low (Figure 5). The total fatty acid was the highest in HSA10 (18.8 mg/g HSA) followed by HSA2 (5.6 mg/g HSA) and then HSA1 (3.7 mg/g HSA). Fatty acid contents of HSA3–6 were relatively similar (around 2 mg/g HSA). These data demonstrate major variations in the fatty acid content of HSA preparations.

**Figure 5:**
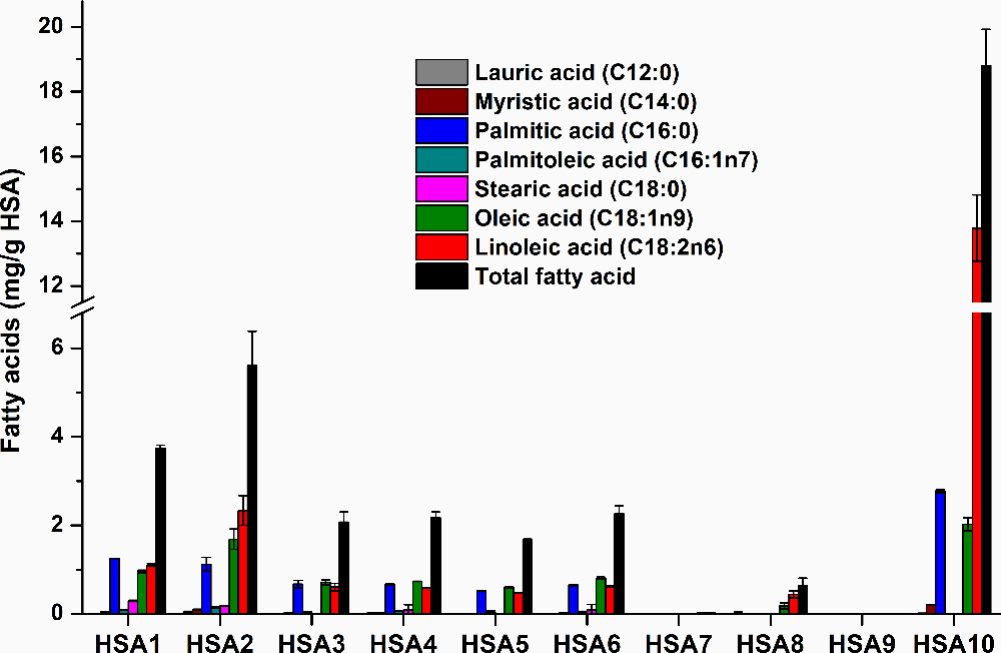
Fatty acid content of HSA preparations (see statistical evaluations for palmitic acid, oleic acid, linoleic acid, and total free fatty acid in Tables S5, S6, S7, and S8, respectively), where total fatty acid means the sum of the seven fatty acids quantified. Fatty acid levels are also demonstrated in separate panels in Figure S1.

### 3.6 Fluorescence Emission Spectra

Fluorescence emission spectra of HSA (2 μM) were recorded, exciting the Trp-214 at 295 nm. Most of the samples showed their emission wavelength maxima around 340 nm, while HSA6 and HSA3 had emission maxima approximately at 345 and 355 nm, respectively (Figure 6). In addition, among the HSA preparations, the emission signals of HSA6 and HSA3 proved to be higher, where the latter sample exerted considerably stronger fluorescence compared to the other albumins tested. HSA2 showed the lowest emission signal.

**Figure 6:**
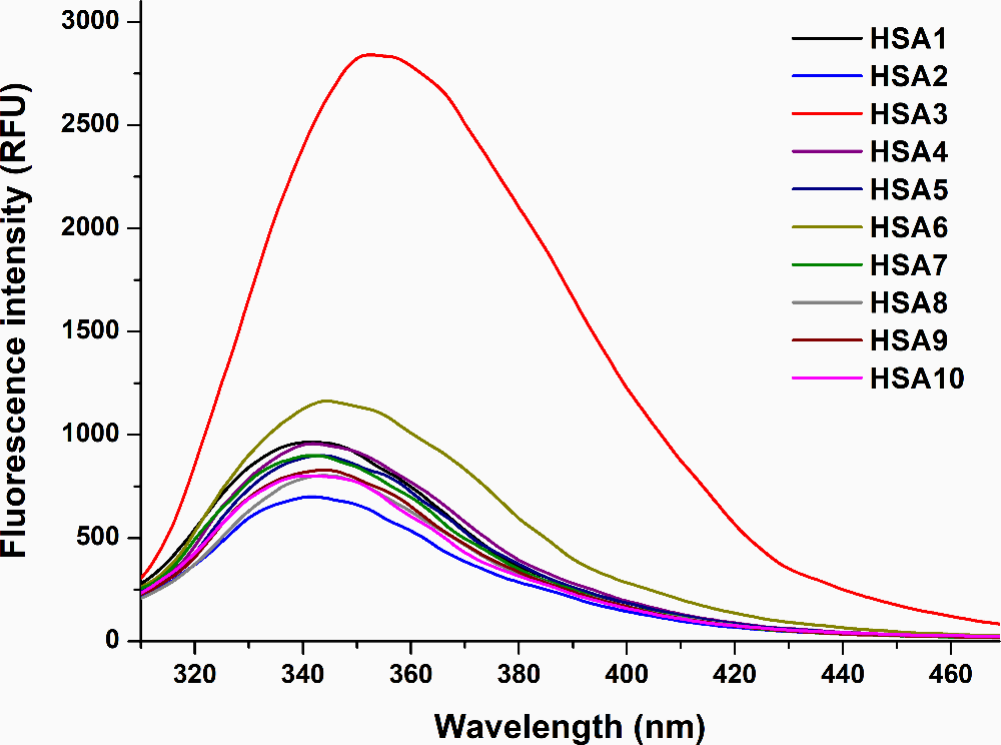
Fluorescence emission spectra (background-corrected) of HSA solutions (2 μM) in PBS (pH 7.4; λ_ex_ = 295 nm; ex slit: 5 nm, em slit: 5 nm).

### 3.7 Fluorescence Quenching Studies

In quenching experiments, increasing concentrations of site I (furosemide and iodipamide), site II (diazepam and ketoprofen), and heme site (biliverdin and methyl orange) markers were added to standard amounts of albumins, and then the quenching effects were evaluated at 340 nm (see representative fluorescence emission spectra in Figure S2). Data represent the relative changes in the emission signal of albumins compared to the emission intensity of HSA in the absence of site markers (Figure 7).

**Figure 7:**
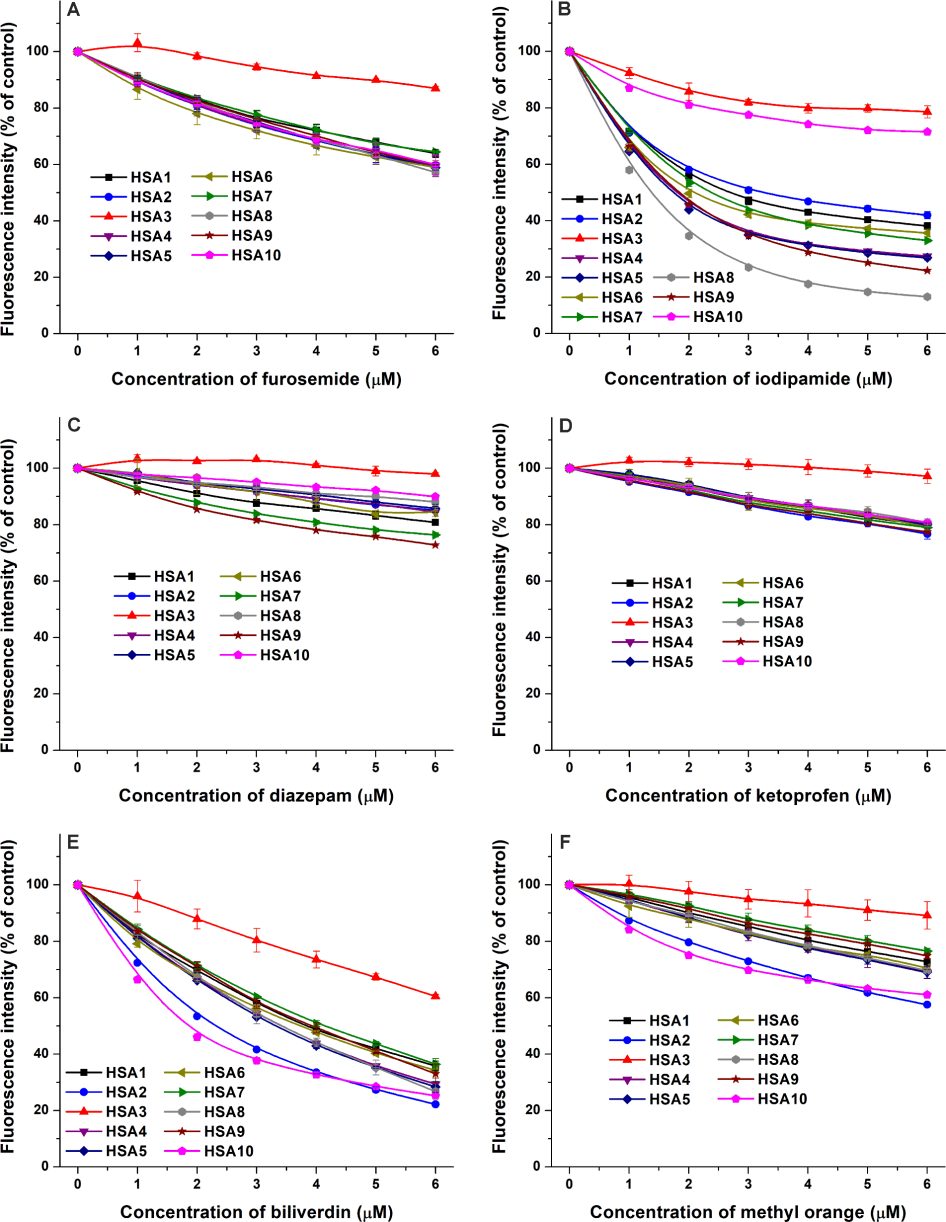
Quenching effects of site I (furosemide and iodipamide), site II (diazepam and ketoprofen), and heme (FA1) site (biliverdin and methyl orange) markers on albumins. Concentration-dependent changes in the emission signals of HSA (2 μM) in the presence of increasing concentrations (0–6 μM) of furosemide (A), iodipamide (B), diazepam (C), ketoprofen (D), biliverdin (E), and methyl orange (F). The relative changes in the emission signal of albumins are demonstrated, where 100% means the emission intensity of HSA without the ligands (λ_ex_ = 295 nm, λ_em_ = 340 nm). Under the applied conditions, site markers did not show background fluorescence in the absence of the protein.

Except HSA3 (where lower quenching effects were noticed), furosemide (site I) induced very similar relative decreases in the emission signal of albumins (Figure 7A). The *K*_SV_ value of furosemide–HSA3 was approximately fivefold lower vs the other furosemide–albumin complexes (Table 2). Interestingly, the quenching effects of the other site I marker drug iodipamide showed large variations: we observed considerably weaker decreases in the emission signals of HSA3 and HSA10, while the strongest quenching was noticed with HSA8 (Figure 7B). The *K*_SV_ value of the iodipamide–HSA8 complex (1.1 × 10^6^ L/mol) was approximately 20-fold and 15-fold higher compared to iodipamide–HSA3 (*K*_SV_ = 5.5 × 10^4^ L/mol) and iodipamide–HSA10 (*K*_SV_ = 7.9 × 10^4^ L/mol), respectively. We determined *K*_SV_ values between 2.6 × 10^4^ and 6.0 × 10^4^ L/mol for the other seven iodipamide–HSA complexes (Table 2).

**Table 2:** Stern–Volmer Quenching Constants (*K*_SV_) of Ligand–HSA Complexes^a^

	furosemide	iodipamide	diazepam	ketoprofen	biliverdin	methyl orange
	*K*_SV_ (× 10^–5^ L/mol)	*K*_SV_ (× 10^–5^ L/mol)	*K*_SV_ (× 10^–5^ L/mol)	*K*_SV_ (× 10^–5^ L/mol)	*K*_SV_ (× 10^–5^ L/mol)	*K*_SV_ (× 10^–5^ L/mol)
**HSA1**	0.96 ± 0.06	3.06 ± 0.08	0.41 ± 0.02	0.41 ± 0.03	2.78 ± 0.12	0.61 ± 0.07
**HSA2**	1.15 ± 0.07	2.63 ± 0.07	0.30 ± 0.00	0.50 ± 0.01	5.34 ± 0.12	1.24 ± 0.02
**HSA3**	0.23 ± 0.01	0.55 ± 0.04			0.97 ± 0.06	0.25 ± 0.02
**HSA4**	1.15 ± 0.11	4.96 ± 0.10	0.30 ± 0.01	0.40 ± 0.02	3.56 ± 0.11	0.73 ± 0.06
**HSA5**	1.15 ± 0.05	5.10 ± 0.11	0.27 ± 0.01	0.39 ± 0.01	3.69 ± 0.14	0.73 ± 0.03
**HSA6**	1.22 ± 0.11	3.54 ± 0.12	0.34 ± 0.04	0.42 ± 0.04	2.96 ± 0.15	0.69 ± 0.02
**HSA7**	0.95 ± 0.04	3.71 ± 0.00	0.57 ± 0.02	0.45 ± 0.01	2.61 ± 0.10	0.49 ± 0.04
**HSA8**	1.18 ± 0.06	11.23 ± 0.32	0.23 ± 0.02	0.39 ± 0.03	3.80 ± 0.32	0.70 ± 0.00
**HSA9**	1.10 ± 0.01	5.97 ± 0.09	0.67 ± 0.01	0.48 ± 0.01	2.94 ± 0.21	0.54 ± 0.03
**HSA10**	1.11 ± 0.08	0.79 ± 0.01	0.18 ± 0.01	0.39 ± 0.00	5.10 ± 0.13	1.20 ± 0.00

a Diazepam and ketoprofen barely affected the emission signal of HSA3; therefore, we could not determine the *K*_SV_ values of these complexes. The *R* ^2^ values regarding the linear fitting of Stern–Volmer plots were in the following ranges: 0.97–0.99 for furosemide, 0.95–0.99 for iodipamide, 0.96–0.99 for diazepam, 0.97–0.99 for ketoprofen, 0.98–0.99 for biliverdin, and 0.97–0.99 for methyl orange.

The site II marker drugs diazepam (Figure 7C) and ketoprofen (Figure 7D) caused negligible decreases in the emission signal of HSA3; therefore, we did not determine the *K*_SV_ value for these complexes. For other albumins tested, we noticed moderate quenching effects of diazepam, where *K*_SV_ values were in the 1.8 × 10^4^ to 6.7 × 10^4^ L/mol range (Table 2). The largest changes were observed with HSA9 and HSA7 (Figure 7C). Except HSA3, ketoprofen caused similar quenching effects on albumins; the *K*_SV_ values were in the 3.9 × 10^4^ to 5.0 × 10^4^ L/mol range (Table 2).

Biliverdin (heme site) moderately reduced the fluorescence signal of HSA3 while it strongly decreased the emission intensities of other albumins examined (Figure 7E). Similar quenching effects and *K*_SV_ values (2.6 × 10^5^ to 3.8 × 10^5^ L/mol) were noticed for HSA1, HSA4, HSA5, HSA6, HSA7, HSA8, and HSA9 (Table 2). Furthermore, somewhat stronger impacts were observed with HSA2 and HSA10 (Figure 7E). We found a more than fivefold difference between the highest (biliverdin–HSA2) and lowest (biliverdin–HSA3) *K*_SV_ values (Table 2). The other heme site marker methyl orange showed somewhat weaker quenching effects than biliverdin, but very similar tendencies were noticed with the lowest impact on HSA3 and the largest changes with HSA2 and HSA10 (Figure 7F). Again, an approximately fivefold difference was observed between the highest (methyl orange–HSA2) and the lowest (methyl orange–HSA3) *K*_SV_ values determined (Table 2).

### 3.8 Ultrafiltration Studies

Since albumin can entrap the bound molecules in the retentate, the ultrafiltration technique is suitable to examine the interaction of HSA with site markers: A stronger interaction of the ligand with the protein results in its lower levels in the filtrate. We tested the impacts of albumins on the filtered fraction of warfarin (site I) and naproxen (site II). The proteins were added to standard amounts of the site markers. After ultrafiltration (with 30 kDa filters), ligand concentrations were analyzed in the filtrate.

HSA1, HSA3, HSA4, HSA5, HSA6, and HSA8 caused very similar decreases in the filtered fraction of warfarin, and we noticed somewhat lower values in the presence of HSA7 and HSA9 (Figure 8A). However, warfarin levels in the filtrate were considerably higher when HSA2 and HSA10 were applied.

**Figure 8:**
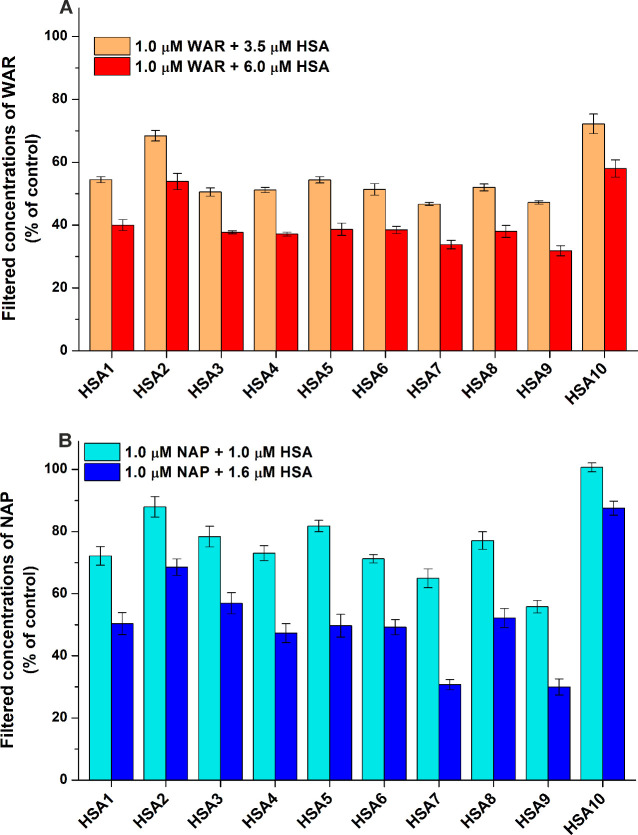
Effects of HSA preparations on the filtered concentrations of the site I marker warfarin (A) and the site II marker naproxen (B). To test the interaction of HSA with site I, we filtered the samples contained warfarin (WAR; 1.0 μM) without or with HSA (3.5 or 6.0 μM) in PBS (pH 7.4). To examine the interaction of HSA with site II, we filtered the samples contained naproxen (NAP; 1.0 μM) in the absence or in the presence of HSA (1.0 or 1.6 μM) in PBS. The concentrations of WAR and NAP in the filtrates were compared to the filtered concentrations of the corresponding site marker without albumin (100%). Statistical evaluations are demonstrated in Tables S9 and S10 for WAR, while in Tables S11 and S12 for NAP.

Interestingly, with the site II marker naproxen, we had similar observations to those with warfarin. At both concentrations tested, HSA1, HSA3, HSA4, HSA5, HSA6, and HSA8 induced comparable changes in the filtered concentration of naproxen (Figure 8B). However, lower levels of the site marker were quantified in the presence of HSA7 and HSA9, while higher concentrations were noticed in the filtrate when HSA2 and HSA10 were presented.

## 4 Discussion

Several studies investigate the interactions of different ligand molecules with HSA, where the data reported sometimes show very large discrepancies. Among the possible reasons, the variations in the quality/purity of HSA preparations can be an explanation. Therefore, to examine this aspect, 10 commercially available HSA preparations were obtained and tested for HSA content, fructosamine level, free thiol concentration, antioxidant capacity, free fatty acid content, fluorescence emission spectra, impacts of site markers (site I, site II, and heme site) on the emission signals of the proteins, and interactions with site I and site II ligands based on ultrafiltration experiments.

The bromocresol green colorimetric method showed similar albumin content of HSA preparations (Figure 1A), suggesting a slightly lower value only for HSA10 (recombinant albumin). The bromocresol green assay is frequently applied in the standard laboratory practice; nevertheless, we need to mention some limitations. Nonspecific interactions can occur with the protein contaminants of recombinant HSA preparations and/or with other plasma proteins (e.g., α_1_- and α_2_-globulins), which may lead to the false-positive overestimation of HSA concentrations.^28,29^ Furthermore, the recombinant origin as well as the presence of free fatty acids (or other high-affinity ligands) can induce slight conformational changes in HSA, which may affect the interaction of the protein with the bromocresol green dye.^30,31^ Interestingly, HSA10 showed similar interactions with furosemide, diazepam, and ketoprofen based on quenching studies (Figure 7A,C,D), slightly stronger interactions with biliverdin and methyl orange based on quenching studies (Figure 7E,F), while weaker interactions with iodipamide (quenching study; Figure 7B), warfarin, and naproxen (ultrafiltration studies; Figure 8) when compared to most of the other proteins examined. SDS-PAGE showed the dominant bands between the 50 and 75 kDa molecular weight markers (Figure 1B), which is in agreement with the molecular weight of HSA (66.5 kDa). Although it is not suitable for quantitative analyses, some differences (amount/size) can be noticed regarding the contaminants with larger molecular weights.

Fructosamine, typically refers to glycated serum proteins, is spontaneously formed by the free amino groups (in lysine and arginine) of serum proteins and glucose in a nonenzymatic process.^32^ After the Schiff base formation of the aldehyde group of the carbohydrate (open/acyclic form) with the amino group of the protein, the intermediate can undergo Amadori rearrangement, leading to the production of a fructosamine derivative. Glycation results in numerous structural and functional changes in HSA, including ligand binding affinity.^33,34^ Nevertheless, depending on the extent of glycation and the ligand applied various changes were noticed, where the stability of ligand–HSA complexes can be increased, decreased, or even unchanged.^33,34^ Normal fructosamine levels of clinical serum samples in nondiabetic individuals are typically in the 200–285 μM range. In our HSA solutions (40 mg/mL), fructosamine levels were between 43 and 284 μM (Figure 2), where we noticed by far the highest level in HSA2. In ultracentrifugation studies, lower binding ability of HSA2 was observed regarding warfarin and naproxen compared to most of the other albumins examined (Figure 8). Nevertheless, we cannot find a clear correlation between these data because several other factors (e.g., protein contaminants, methionine oxidation, protein carbonylation, or protein aggregation) may affect the interaction of these ligands with HSA2.

Cys-34 represents the only free thiol group of HSA, approximately 70–80% of albumin molecules represent Cys-34 as a free sulfhydryl group in healthy adults (the other 20–30% form mixed disulfides typically with cysteine, homocysteine, or glutathione).^1^ Considering the HSA content of our solutions tested (40 mg/mL ≈ 600 μM albumin), free thiol levels were less than 30% because the highest free thiol concentration determined was only 173 μM (HSA1). Among the albumins tested, relatively higher free thiol concentrations were observed in HSA1, HSA7, HSA8, HSA9, and HSA10 (134–173 μM), while lower levels were noticed in HSA2, HSA3, HSA4, HSA5, and HSA6 (55–97 μM). Furthermore, Cys-34 works as a physiological antioxidant, taking part in radical scavenging.^1^ Despite the marked differences noticed in the free thiol levels of HSA preparations (Figure 3), HSA1–9 showed similar antioxidant capacity (Figure 4). Interestingly, the recombinant albumin (HSA10) had a considerably stronger antioxidant effect compared to the other proteins examined.

The free fatty acid content of HSA preparations showed marked variations (Figure 5 and Figure S1). It is not surprising that no or negligible amounts of fatty acids were detected in HSA7 and HSA9, because these were sold as fatty acid free proteins (Table 1). The fatty acid content of HSA8 (the globulin free product) was also low (Figure 5). In contrast, exceptionally high fatty acid levels were noticed in HSA10 (human recombinant albumin, expressed in rice), and HSA2 also had relatively high fatty acid content compared to the other proteins. The free fatty acid level and composition were similar in HSA3, HSA4, HSA5, and HSA6, while somewhat higher amounts were noticed in HSA1 (Figure 5). Interestingly, HSA2 and HSA10 (with high fatty acid content) showed weaker interactions with warfarin and naproxen in ultrafiltration studies (Figure 8) and formed more stable complexes with biliverdin and methyl orange in quenching studies (Figure 7E,F) compared to the other proteins. In contrast, HSA7 and HSA9 (with close to zero fatty acid levels) represented somewhat stronger binding ability regarding warfarin and naproxen (Figure 8). These data may suggest the correlation of fatty acid content and ligand binding, but it seems to be controversial based on other details, as we will discuss it later.

Fluorescence spectroscopy is a relatively cheap and widely available technique; therefore, it is commonly used to investigate ligand–albumin interactions. Furthermore, fluorescence quenching is one of the most commonly applied methods in scientific articles to evaluate the interaction of ligand molecules with albumin,^13,14^ where the ligand-induced decreases in the emission signal of HSA are evaluated. HSA contains only one tryptophan moiety at position 214 (Trp-214) in subdomain IIA (site I), which has the largest role in the fluorescence emission signal of the protein (with moderate and low involvement of tyrosine and phenylalanine amino acids, respectively).^1^ Importantly, besides the complex formation, other processes (e.g., collisional quenching and/or Förster resonance energy transfer) may also affect the emission signal of HSA.^13^ Emission spectra of HSA3 showed marked differences compared to the other albumins examined (Figure 6). The bromocresol green colorimetric method suggested the same albumin content of HSA3 as the other proteins (Figure 1A); thus, it is likely caused by some pollutant(s) in this preparation with strong fluorescence property. This hypothesis was also supported by our observations that the ligand-induced relative changes in the emission signal of HSA3 were lower compared to the other albumins (Figure 7), while the binding ability of HSA3 was similar to HSA1, HSA4, HSA5, HSA6, and HSA8 in ultrafiltration experiments (Figure 8). To confirm the presence of small fluorophore molecule(s), HSA3 was ultrafiltered through 30 kDa filters in PBS and in the 25 v/v% isopropanol–PBS mixture. In another experiment, a twofold volume of acetonitrile was added to the protein solution, after which the precipitated albumin was centrifuged. Then, the fluorescence emission spectra of the filtrates and the supernatants were collected and compared. Even the simple ultrafiltration of HSA3 (in PBS) resulted in the appearance of a significant emission peak around 355 nm in the filtrate (Figure S3). The intensity of this signal was further increased when HSA3 was filtered with isopropanol or the protein was precipitated with acetonitrile (Figure S3). During the latter two treatments, the protein was not simply washed by the solvent but the ligand–protein complexes were disrupted by isopropanol and acetonitrile. These observations proved that small fluorophore pollutant(s) are contained by HSA3. Thus, the quenching studies with HSA3 were compromised, due to the other fluorophore(s) that partly covered the ligand-induced quenching effects. The fluorescence emission spectrum of HSA6 also showed some differences compared to the other proteins (Figure 6); however, in quenching studies, it behaved pretty similarly to most of the other proteins with each ligand examined (Figure 7).

As an interesting observation in quenching studies, the site marker selected has a high importance. Among the site I markers, very similar quenching effects were noticed with furosemide, while major variations were found with iodipamide (Figure 7A,B). Regarding the site II markers, we noticed only minor differences with ketoprofen, while larger discrepancies were observed with diazepam (Figure 7C,D). However, the heme site markers biliverdin and methyl orange showed similar tendencies (Figure 7E,F). In addition, other site I (warfarin) and site II (naproxen) markers showed stronger (HSA7 and HSA9) or weaker (HSA2 and HSA10) interactions with certain proteins in ultrafiltration experiments (Figure 8).

The ligand binding properties of HSA1, HSA3, HSA4, HSA5, HSA6, and HSA8 seem to be similar. Nevertheless, HSA3 provided misleading results in quenching studies. HSA7 and HSA9 showed somewhat stronger interactions with diazepam (Figure 7C and Table 2), warfarin and naproxen (Figure 8). Furthermore, HSA2 and HSA10 demonstrated their stronger interactions with biliverdin and methyl orange (Figure 7E,F) as well as the formation of their less stable complexes were observed with warfarin and naproxen (Figure 8). In addition, the relatively weak interaction of HSA10 with iodipamide can also be noticed (Figure 7B). As mentioned above, we could see some correlations between fatty acid content and ligand binding ability, because HSA7 and HSA9 are fatty acid free preparations, while we detected high fatty acid levels in HSA2 and HSA10. Earlier reports emphasized that fatty acids can affect the binding affinity of other ligands toward HSA.^35–37^ Nevertheless, previous reports clearly demonstrated that the presence of different fatty acids (including palmitic acid, stearic acid, oleic acid, or linoleic acid) results in the stronger interaction of warfarin with HSA.^12,37^ Therefore, it is surprising that in our current investigation fatty acid free proteins (HSA7 and HSA9) showed slightly stronger interactions with warfarin, while HSA2 and HSA10 (with high fatty acid contents) demonstrated weaker complex formation (Figure 8A). In addition, the fatty acid content of HSA8 was low (Figure 5), but it was not accompanied by its stronger interactions with warfarin or naproxen (Figure 8). Thus, we do not see clear evidence that the fatty acid content of HSA preparations is responsible for the discrepancies in their binding affinity.

Interestingly, the antioxidant capacity of HSA preparations was very similar (except HSA10); however, other parameters (e.g., fructosamine, free thiol, and fatty acid levels) showed large variations, sometimes with several-fold discrepancies. Despite the differences of HSA preparations (e.g., normal, fatty acid free, and globulin free products), bromocresol green colorimetric assay suggested similar albumin contents. Nevertheless, electropherograms as well as the much higher emission signal of HSA3 (compared to the other preparations) highlight the presence and importance of some pollutants (e.g., other proteins and/or small ligands). It is also important to emphasize that the same HSA can behave similarly or even can show considerable differences in its ligand binding ability vs the other albumin preparations, depending on the binding site involved and/or the site marker applied in the assay.

As limitations of our study, we would like to mention the following aspects: We did the comparison of 10 commercially available HSA preparations; however, the current study did not examine the potential lot-to-lot variations. We tested several properties/interactions of the 10 HSA preparations obtained; nevertheless, numerous other interesting issues can be considered (including the detailed analyses of contaminants/impurities, protein aggregation, and oxidation and/or carbonylation of albumin), which were not investigated in the present study.

In conclusion, our study describes the complex investigation and comparison of 10 HSA preparations (including fatty acid free, globulin free, and recombinant products), providing novel data regarding the possible differences. These results highlight that sometimes even major variations in certain properties/interactions can be observed. Therefore, the precise specification and the careful selection seem to be reasonable. Based on our results, HSA1, HSA4, HSA5, HSA6, and HSA8 seem to be proper albumin preparations for the investigation of ligand–HSA interactions; nevertheless, we cannot provide strong recommendations because of the possible lot-to-lot variations.

## Supplementary Material



## Notes

Detailed description of the experimental regarding electrophoresis; lauric acid, myristic acid, palmitic acid, palmitoleic acid, stearic acid, oleic acid, linoleic acid, and total fatty acid contents (mg fatty acid/g protein) of the HSA preparations examined (Figure S1); representative fluorescence emission spectra of HSA preparations in the presence of increasing concentrations of site markers (Figure S2); fluorescence emission spectra of HSA3 vs the emission signals of ultrafiltrates (PBS or 25 v/v% of isopropanol) and the supernatant (after addition of twofold volume of acetonitrile and centrifugation) (Figure S3); results of the statistical analyses (one-way ANOVA and Tukey’s post hoc tests; *p* < 0.01) (Tables S1–S12) (PDF)
