# Supplementary material for: Complex Investigation of the Similarities and Differences between Ten Commercially Available Human Serum Albumin Preparations
Source: ACS Omega. 2025 Sep 25;10(39):45883–93. doi: 10.1021/acsomega.5c06541 (PMC12509120; doi:10.1021/acsomega.5c06541)
Supplement: Supplementary file 1 [file ao5c06541_si_001.pdf]

Complex investigation of the similarities and the differences between ten commercially available human serum albumin preparations

SUPPLEMENTARY MATERIAL

Rita Jakabfi-Csepregi [1,2], Zoltán Nagymihály [2], Zoltán Horváth-Szalai [1,2], Balázs Szirmay [1], Éva Varga-Visi [3], Omeralfaroug Ali [3], Edward Agyarko [3], András Szabó [3,4], Miklós Poór [1,2,*]

[1] Department of Laboratory Medicine, Medical School, University of Pécs, Ifjúság útja 13, H-7624 Pécs, Hungary

[2] Molecular Medicine Research Group, János Szentágothai Research Centre, University of Pécs, Ifjúság útja 20, H-7624 Pécs, Hungary

[3] Agribiotechnology and Precision Breeding for Food Security National Laboratory, Institute of Physiology and Nutrition, Department of Physiology and Animal Health, Hungarian University of Agriculture and Life Sciences, Guba Sándor u. 40, H-7400 Kaposvár, Hungary

[4] HUN-REN-MATE Mycotoxins in the Food Chain Research Group, Hungarian University of Agriculture and Life Sciences, Guba Sándor u. 40, H-7400 Kaposvár, Hungary

*Corresponding author: Miklós Poór, PharmD, PhD
Department of Laboratory Medicine,
Medical School, University of Pécs
Ifjúság útja 13, H-7624 Pécs, Hungary
Phone: +36-72-501-500 ext: 29250
E-mail: poor.miklos@pte.hu

Electrophoresis

Gel slabs for electrophoresis were manually prepared by using Mini-Protean Glass Plates with 0.75 mm spacers (Bio-Rad) in casting stands in the Department of Laboratory Medicine (University of Pécs, Medical School, Hungary). To prepare two pieces of separating gels (9 × 12 cm), the following chemicals/solutions were used: 4.2 mL distilled water, 3.4 mL solution A (30% acrylamide and 0.8% N,N-methylene-bis-acrylamide), 2.5 mL solution B (18.17 g Tris dissolved in distilled water then adjusted with 6 M HCl to pH 8.8, after which 4 mL of 10% SDS solution was added, and finally made up to 100 mL with distilled water), 30 µL of freshly prepared solution F (100 mg/mL ammonium persulphate), and 5 µL of TEMED. Polymerization time regarding separating gels took 2 h. For preparing two pieces of stacking gels, the following solutions were applied: 2.9 mL distilled water, 0.83 mL solution A, 1.25 mL solution C (6.06 g Tris dissolved in distilled water then adjusted with 6 M HCl to pH 6.8, after which 4 mL of 10% SDS was added, and finally made up to 100 mL with distilled water), 15 µL solution F, and 5 µL TEMED. The stacking gel solution was cast onto the top of the polymerized separating gels, then Mini-Protean electrophoresis combs (10 wells; Bio-Rad) were gently placed into the stacking gel solution and the polymerization was allowed to proceed for 1.5 h. After loading of HSA samples (20 µg protein/well) and the molecular weight marker, electrophoresis was performed at 150 V for 1 h applying a Tris-glycine solution containing 0.1% SDS as running buffer.

After electrophoretic analysis, gels were placed into CBB staining solution (1 g CBB, 450 mL distilled water, 450 mL methanol, and 100 mL glacial acetic acid) for 2 h to visualize individual protein bands. Thereafter, gels were washed multiple times with differentiation solution (100 mL glacial acetic acid, 450 mL methanol, and 450 mL distilled water) until the desired background and band intensities in the gels were achieved. Then the gels were stored in a 10% acetic acid solution.

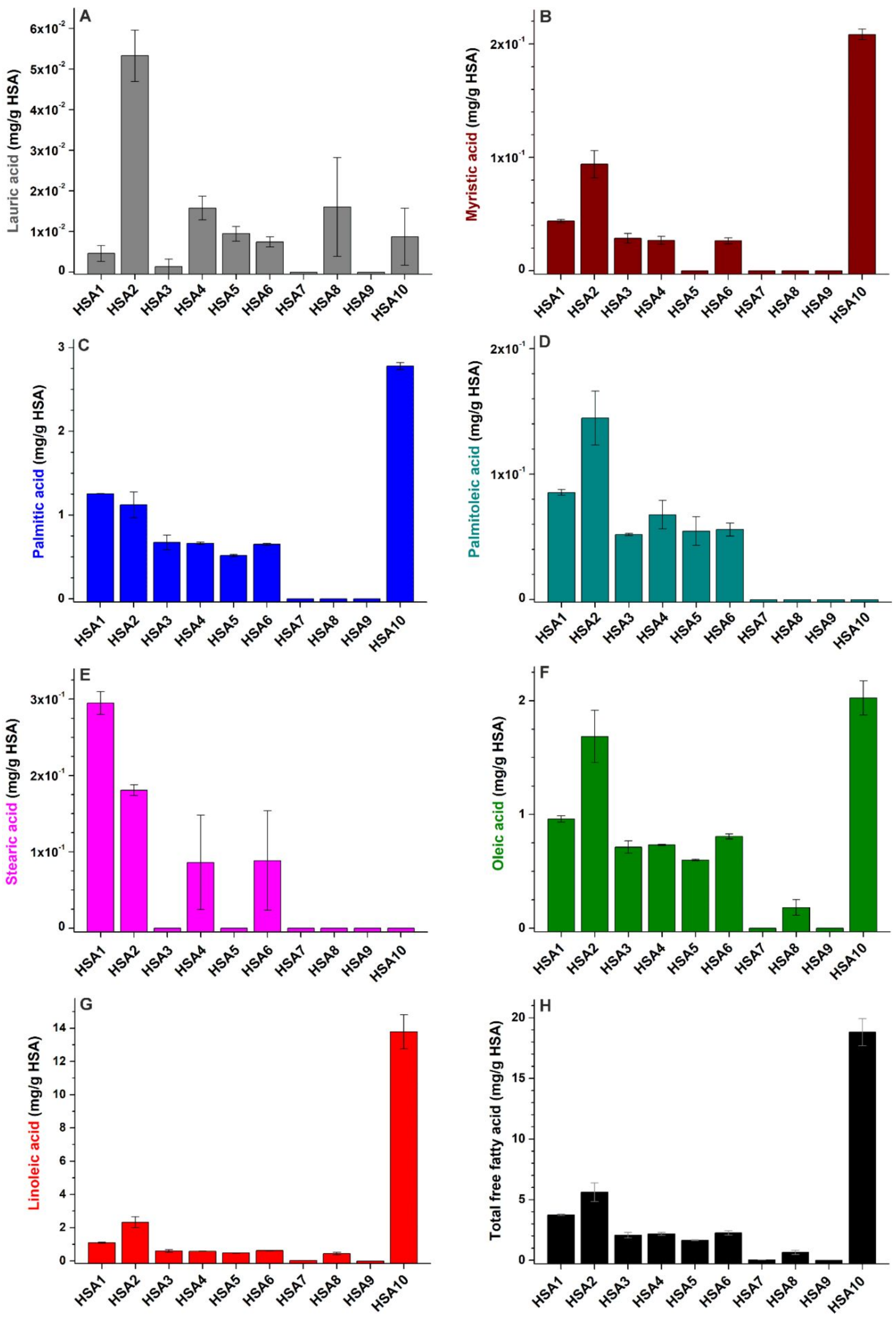

Fig. S1: Free fatty acid content (mg fatty acid/g protein) of the HSA preparations examined: lauric acid (**A**), myristic acid (**B**), palmitic acid (**C**), palmitoleic acid (**D**), stearic acid (**E**), oleic acid (**F**), linoleic acid (**G**), and the sum of the seven fatty acids listed (**H**; total free fatty acid).

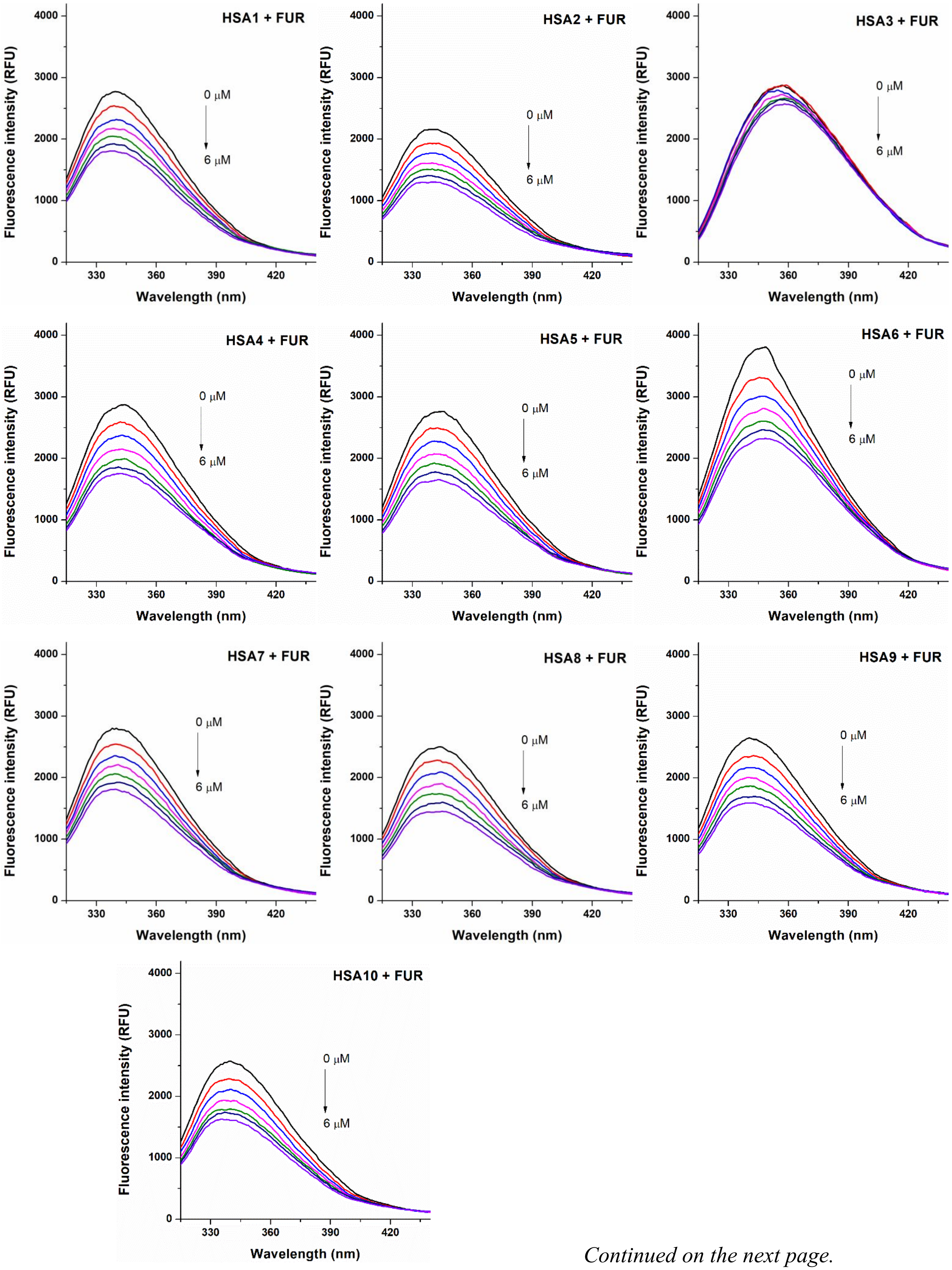

Continued on the next page.

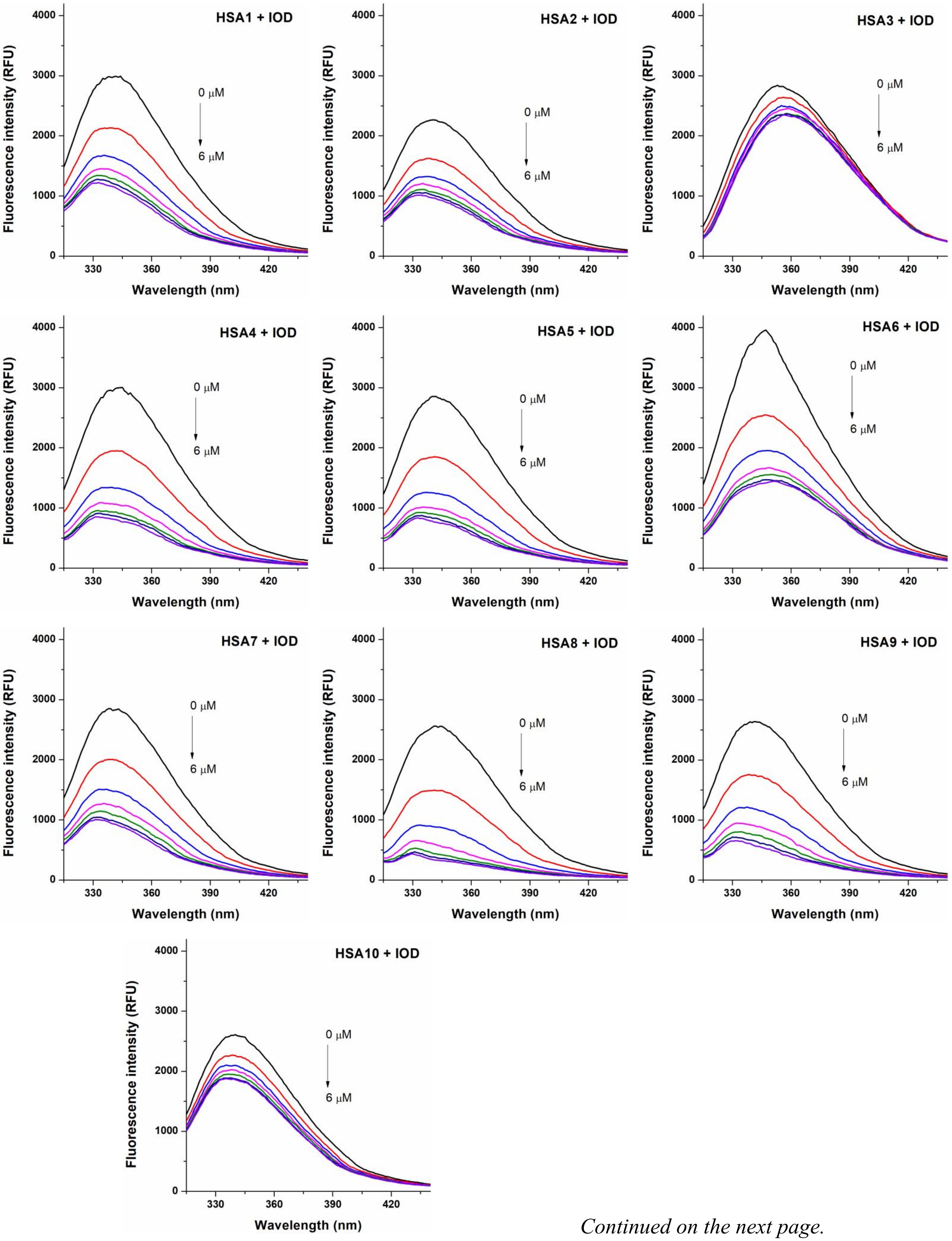

Continued on the next page.

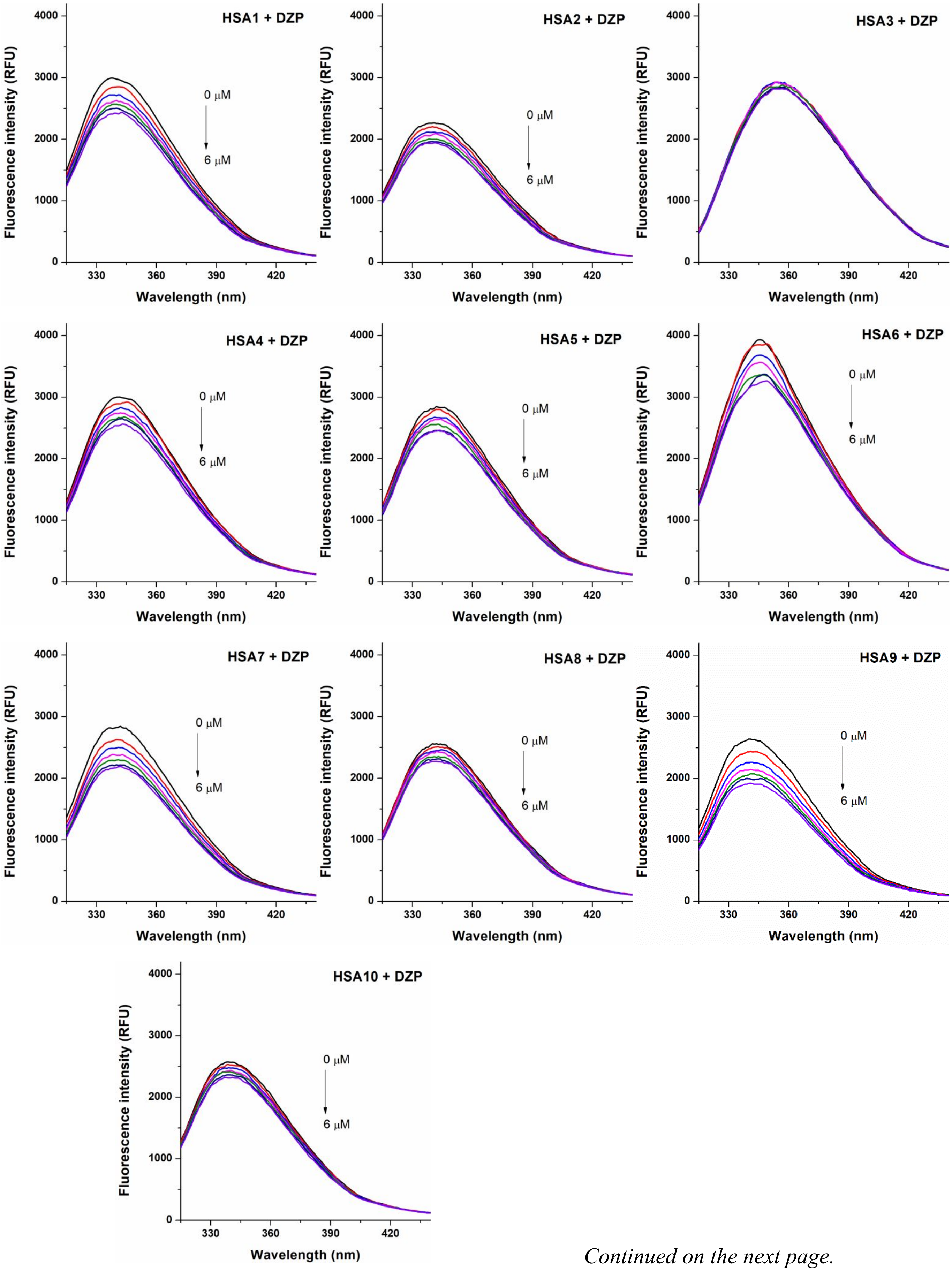

Continued on the next page.

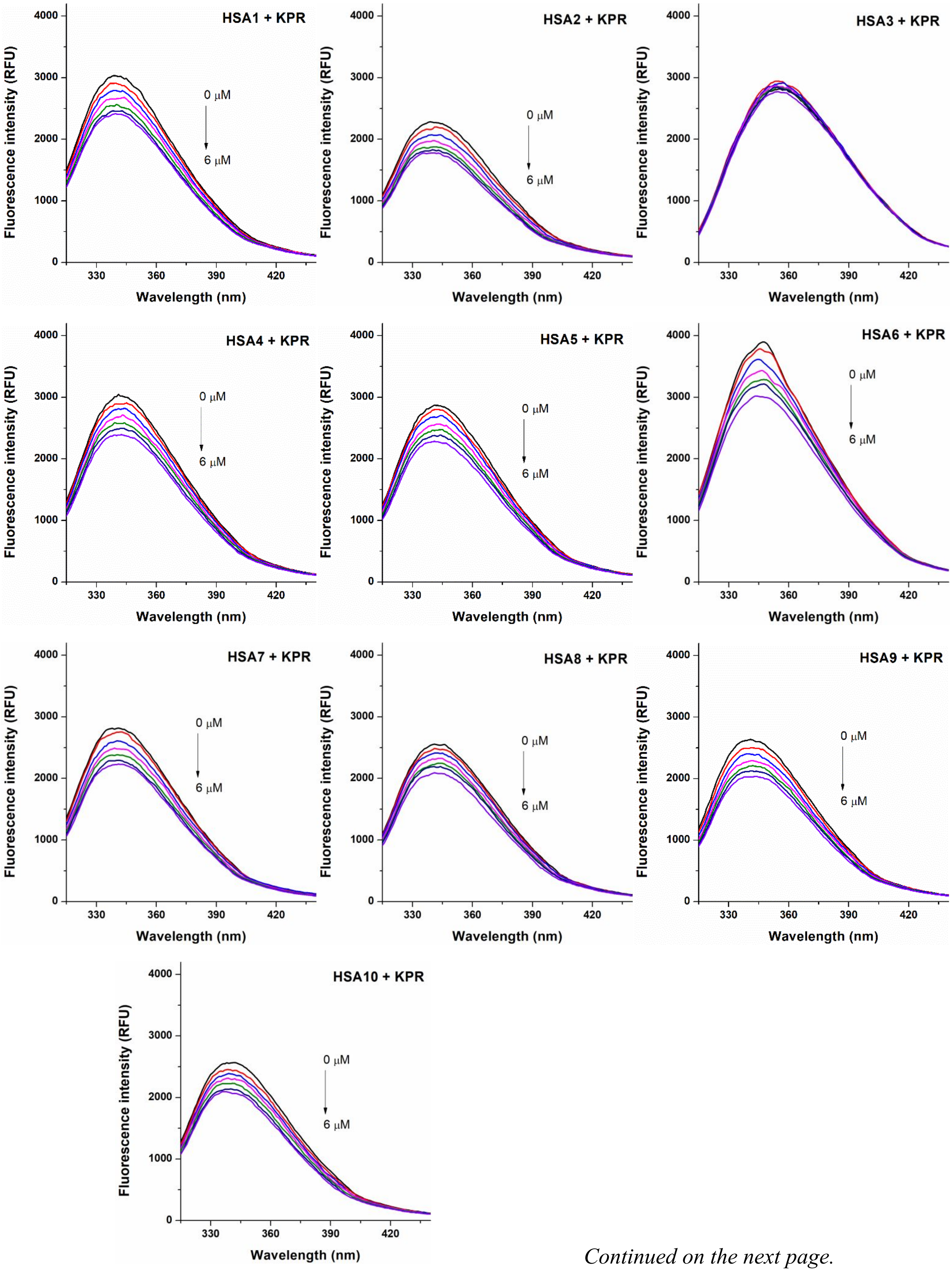

Continued on the next page.

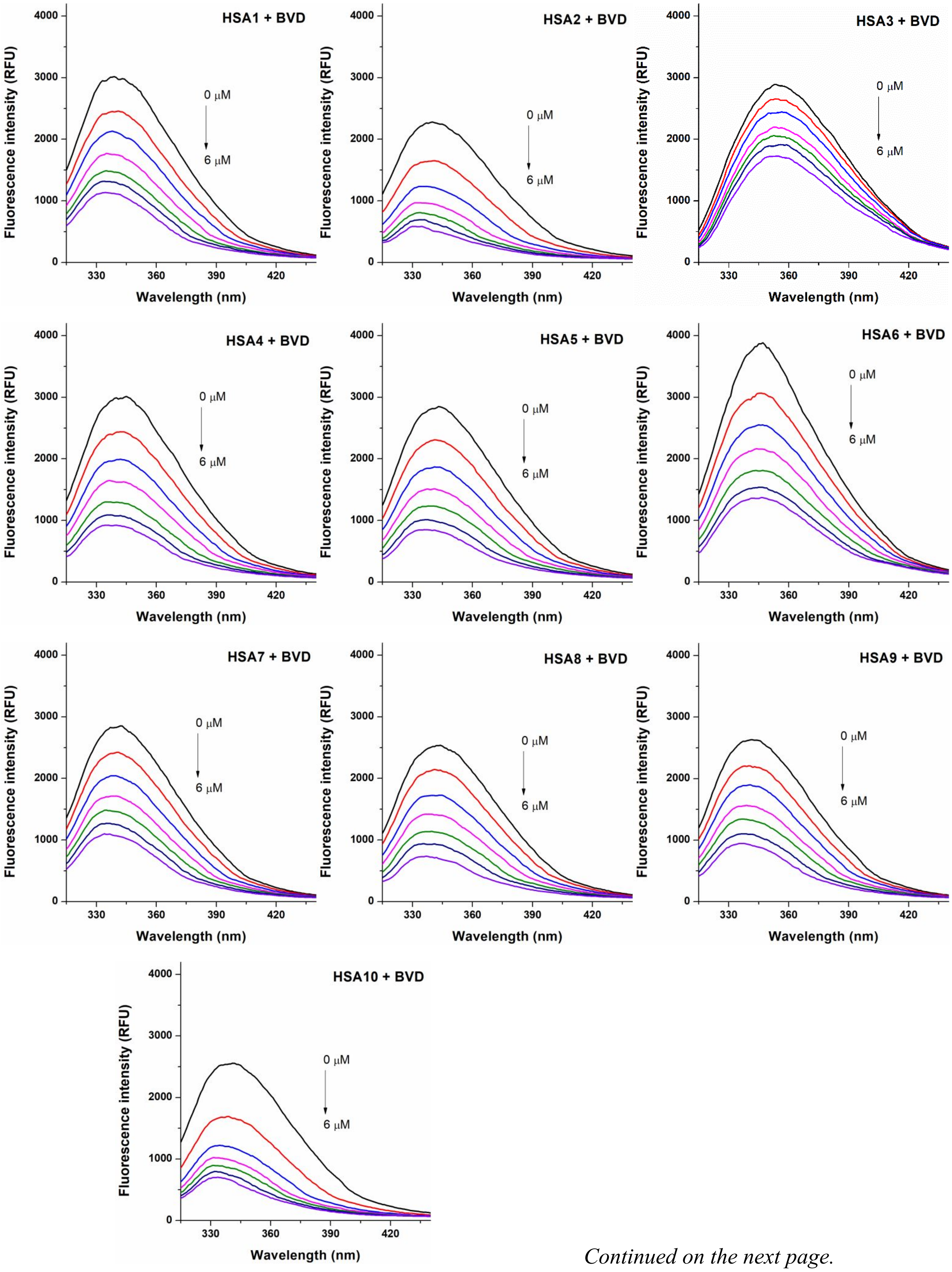

Continued on the next page.

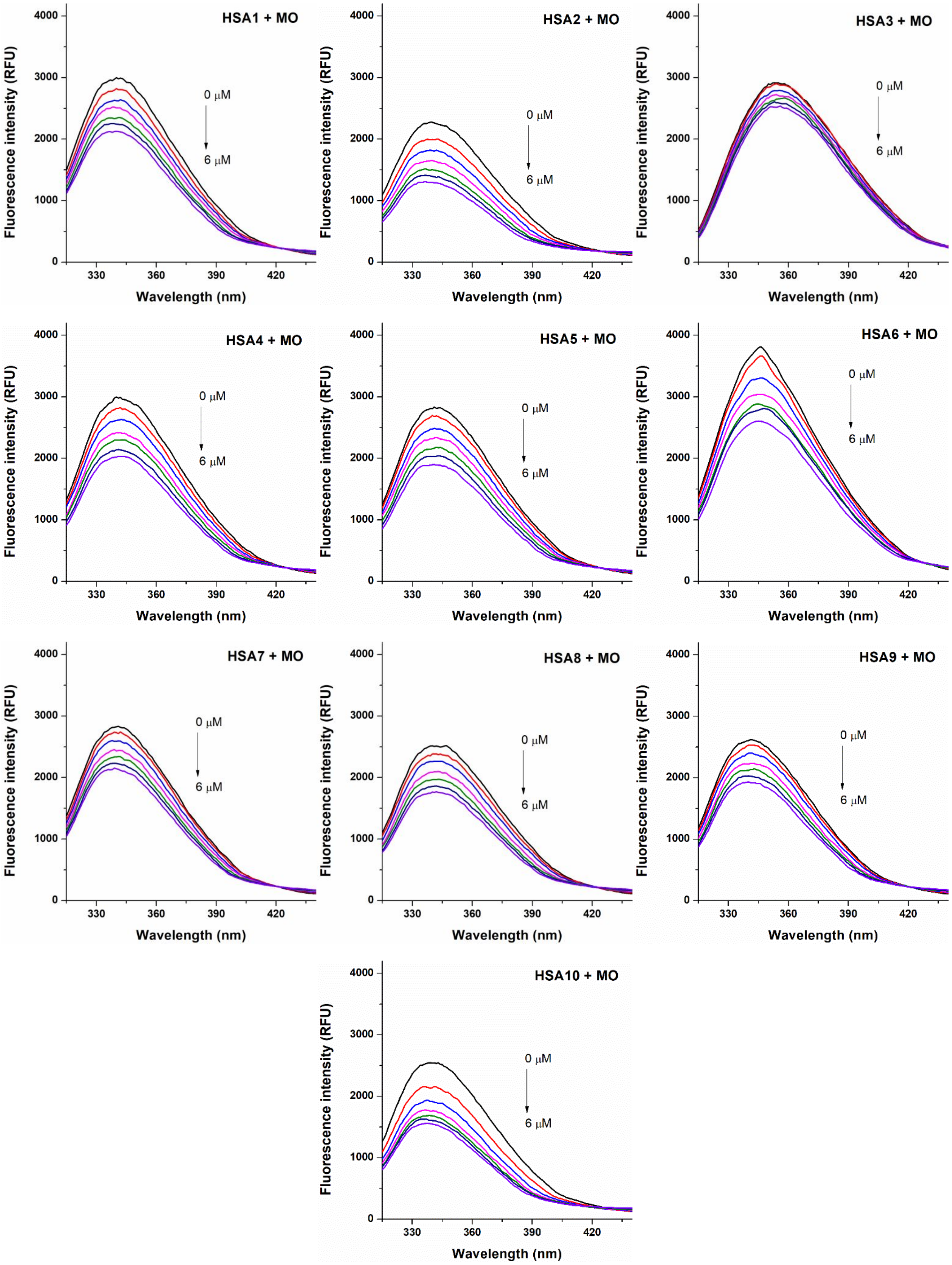
HSA1 + MO
HSA2 + MO
HSA3 + MO
HSA4 + MO
HSA5 + MO
HSA6 + MO
HSA7 + MO
HSA8 + MO
HSA9 + MO
HSA10 + MO
0 μM
6 μM
Fluorescence intensity (RFU)
Wavelength (nm)
0
1000
2000
3000
4000
330
360
390
420

Fig. S2: Representative fluorescence emission spectra of HSA preparations in the presence of increasing concentrations of site markers (FUR, furosemide; IOD, iodipamide; DZP, diazepam; KPR, ketoprofen; BVD, biliverdin; MO, methyl orange) in PBS (pH 7.4; HSA concentration: 2 μM; ligand concentrations: 0, 1, 2, 3, 4, 5, and 6 μM; λ_{ex} = 295 nm). Except HSA3, the ex and em slits were 5 nm and 10 nm, respectively. Due to its considerably higher emission signal compared to the other albumins, in the measurements with HSA3, both ex and em slits were 5 nm.

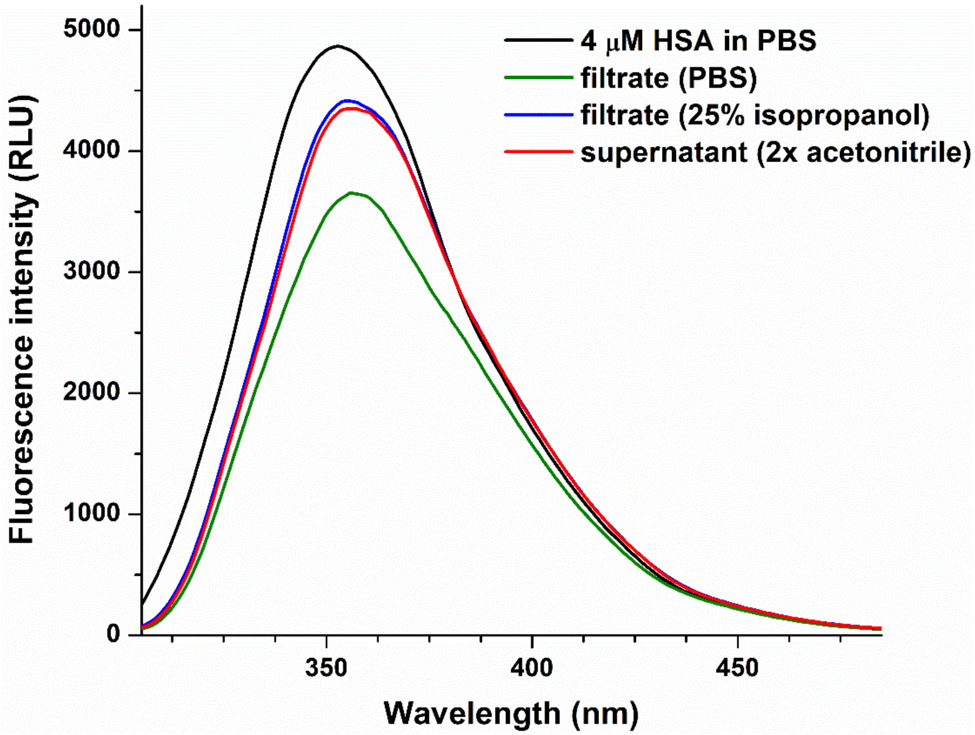

Fig. S3: Fluorescence emission spectra of HSA3 (4 μM) in PBS, and the emission signals measured in the filtrates and in the supernatant (λ_{ex} = 295 nm), where samples were diluted according to the nominal 4 μM albumin concentration. Each process described below was also performed with PBS (without the protein), where we did not notice any background signal.

In ultrafiltration experiments, a 500 μL volume of 20 μM HSA3 solutions (dissolved in PBS or in 25 v/v% isopropanol–PBS mixture) were filtered (10 min, 7500 g, room temperature) using Amicon Ultra centrifugal filters (30 kDa; Merck, Darmstadt, Germany). Thereafter, the filtrate was homogenized and diluted to fivefold volume with PBS (final isopropanol level ≈ 5 v/v%).

In the other experiment, a 150 μL volume of 240 μM HSA3 solution (dissolved in PBS) was prepared, then 300 μL of acetonitrile was added. After intensive vortexing, the precipitated protein was removed by centrifugation (10 min, 14,000 g, room temperature). Thereafter, the supernatant was diluted to 20-fold with PBS (final acetonitrile level ≈ 3.3 v/v%).

Table S1: Analysis of statistical differences (one-way ANOVA with Tukey's post hoc test) regarding the albumin concentrations determined from 40 g/L HSA samples based on the bromocresol green colorimetric method (see data in Fig. 1A).

HSA levels										
	**HSA1**	**HSA2**	**HSA3**	**HSA4**	**HSA5**	**HSA6**	**HSA7**	**HSA8**	**HSA9**	**HSA10**
**HSA1**	–	n.s.	n.s.	n.s.	n.s.	n.s.	n.s.	n.s.	n.s.	<0.01
**HSA2**	n.s.	–	n.s.	n.s.	n.s.	n.s.	n.s.	n.s.	n.s.	<0.01
**HSA3**	n.s.	n.s.	–	n.s.	n.s.	n.s.	n.s.	n.s.	n.s.	<0.01
**HSA4**	n.s.	n.s.	n.s.	–	n.s.	n.s.	n.s.	n.s.	n.s.	<0.01
**HSA5**	n.s.	n.s.	n.s.	n.s.	–	n.s.	n.s.	n.s.	n.s.	<0.01
**HSA6**	n.s.	n.s.	n.s.	n.s.	n.s.	–	<0.01	n.s.	n.s.	<0.01
**HSA7**	n.s.	n.s.	n.s.	n.s.	n.s.	<0.01	–	n.s.	n.s.	<0.01
**HSA8**	n.s.	n.s.	n.s.	n.s.	n.s.	n.s.	n.s.	–	n.s.	<0.01
**HSA9**	n.s.	n.s.	n.s.	n.s.	n.s.	n.s.	n.s.	n.s.	–	<0.01
**HSA10**	<0.01	<0.01	<0.01	<0.01	<0.01	<0.01	<0.01	<0.01	<0.01	–

n.s. = not significant

Table S2: Analysis of statistical differences (one-way ANOVA with Tukey's post hoc test) regarding the fructosamine levels determined in 40 g/L HSA samples (see data in Fig. 2).

Fructosamine levels										
	**HSA1**	**HSA2**	**HSA3**	**HSA4**	**HSA5**	**HSA6**	**HSA7**	**HSA8**	**HSA9**	**HSA10**
**HSA1**	–	<0.01	<0.01	<0.01	<0.01	<0.01	<0.01	<0.01	<0.01	<0.01
**HSA2**	<0.01	–	<0.01	<0.01	<0.01	<0.01	<0.01	<0.01	<0.01	<0.01
**HSA3**	<0.01	<0.01	–	n.s.	<0.01	n.s.	<0.01	<0.01	<0.01	<0.01
**HSA4**	<0.01	<0.01	n.s.	–	<0.01	n.s.	<0.01	<0.01	<0.01	<0.01
**HSA5**	<0.01	<0.01	<0.01	<0.01	–	<0.01	<0.01	<0.01	<0.01	<0.01
**HSA6**	<0.01	<0.01	n.s.	n.s.	<0.01	–	<0.01	<0.01	<0.01	<0.01
**HSA7**	<0.01	<0.01	<0.01	<0.01	<0.01	<0.01	–	<0.01	<0.01	<0.01
**HSA8**	<0.01	<0.01	<0.01	<0.01	<0.01	<0.01	<0.01	–	<0.01	<0.01
**HSA9**	<0.01	<0.01	<0.01	<0.01	<0.01	<0.01	<0.01	<0.01	–	n.s.
**HSA10**	<0.01	<0.01	<0.01	<0.01	<0.01	<0.01	<0.01	<0.01	n.s.	–

n.s. = not significant

Table S3: Analysis of statistical differences (one-way ANOVA with Tukey's post hoc test) regarding the levels of free thiol groups determined in 40 g/L HSA samples (see data in Fig. 3).

**Free thiol groups**										
	**HSA1**	**HSA2**	**HSA3**	**HSA4**	**HSA5**	**HSA6**	**HSA7**	**HSA8**	**HSA9**	**HSA10**
**HSA1**	–	<0.01	<0.01	<0.01	<0.01	<0.01	<0.01	<0.01	<0.01	<0.01
**HSA2**	<0.01	–	n.s.	<0.01	<0.01	n.s.	<0.01	<0.01	<0.01	<0.01
**HSA3**	<0.01	n.s.	–	<0.01	<0.01	n.s.	<0.01	<0.01	<0.01	<0.01
**HSA4**	<0.01	<0.01	<0.01	–	<0.01	n.s.	<0.01	<0.01	<0.01	<0.01
**HSA5**	<0.01	<0.01	<0.01	<0.01	–	<0.01	<0.01	<0.01	<0.01	<0.01
**HSA6**	<0.01	n.s.	n.s.	n.s.	<0.01	–	<0.01	<0.01	<0.01	<0.01
**HSA7**	<0.01	<0.01	<0.01	<0.01	<0.01	<0.01	–	n.s.	n.s.	n.s.
**HSA8**	<0.01	<0.01	<0.01	<0.01	<0.01	<0.01	n.s.	–	n.s.	n.s.
**HSA9**	<0.01	<0.01	<0.01	<0.01	<0.01	<0.01	<0.01	<0.01	–	<0.01
**HSA10**	<0.01	<0.01	<0.01	<0.01	<0.01	<0.01	n.s.	n.s.	n.s.	–

n.s. = not significant

Table S4: Analysis of statistical differences (one-way ANOVA with Tukey's post hoc test) regarding the total antioxidant capacity of 40 g/L HSA samples (see data in Fig. 4).

**Total antioxidant capacity**										
	**HSA1**	**HSA2**	**HSA3**	**HSA4**	**HSA5**	**HSA6**	**HSA7**	**HSA8**	**HSA9**	**HSA10**
**HSA1**	–	n.s.	<0.01	n.s.	n.s.	n.s.	n.s.	<0.01	n.s.	<0.01
**HSA2**	n.s.	–	n.s.	n.s.	n.s.	n.s.	n.s.	<0.01	n.s.	<0.01
**HSA3**	<0.01	n.s.	–	n.s.	n.s.	n.s.	<0.01	n.s.	n.s.	<0.01
**HSA4**	n.s.	n.s.	n.s.	–	n.s.	n.s.	n.s.	<0.01	n.s.	<0.01
**HSA5**	n.s.	n.s.	n.s.	n.s.	–	n.s.	n.s.	n.s.	n.s.	<0.01
**HSA6**	n.s.	n.s.	n.s.	n.s.	n.s.	–	n.s.	n.s.	n.s.	<0.01
**HSA7**	n.s.	n.s.	<0.01	n.s.	n.s.	n.s.	–	<0.01	n.s.	<0.01
**HSA8**	<0.01	<0.01	n.s.	<0.01	n.s.	n.s.	<0.01	–	<0.01	<0.01
**HSA9**	n.s.	n.s.	n.s.	n.s.	n.s.	n.s.	n.s.	<0.01	–	<0.01
**HSA10**	<0.01	<0.01	<0.01	<0.01	<0.01	<0.01	<0.01	<0.01	<0.01	–

n.s. = not significant

Table S5: Analysis of statistical differences (one-way ANOVA with Tukey's post hoc test) regarding the palmitic acid content of HSA preparations (see data in Fig. 5 and Fig. S1C).

**Palmitic acid content**										
	**HSA1**	**HSA2**	**HSA3**	**HSA4**	**HSA5**	**HSA6**	**HSA7**	**HSA8**	**HSA9**	**HSA10**
**HSA1**	–	n.s.	<0.01	<0.01	<0.01	<0.01	<0.01	<0.01	<0.01	<0.01
**HSA2**	n.s.	–	<0.01	<0.01	<0.01	<0.01	<0.01	<0.01	<0.01	<0.01
**HSA3**	<0.01	<0.01	–	n.s.	n.s.	n.s.	<0.01	<0.01	<0.01	<0.01
**HSA4**	<0.01	<0.01	n.s.	–	n.s.	n.s.	<0.01	<0.01	<0.01	<0.01
**HSA5**	<0.01	<0.01	n.s.	n.s.	–	n.s.	<0.01	<0.01	<0.01	<0.01
**HSA6**	<0.01	<0.01	n.s.	n.s.	n.s.	–	<0.01	<0.01	<0.01	<0.01
**HSA7**	<0.01	<0.01	<0.01	<0.01	<0.01	<0.01	–	n.s.	n.s.	<0.01
**HSA8**	<0.01	<0.01	<0.01	<0.01	<0.01	<0.01	n.s.	–	n.s.	<0.01
**HSA9**	<0.01	<0.01	<0.01	<0.01	<0.01	<0.01	n.s.	n.s.	–	<0.01
**HSA10**	<0.01	<0.01	<0.01	<0.01	<0.01	<0.01	<0.01	<0.01	<0.01	–

n.s. = not significant

Table S6: Analysis of statistical differences (one-way ANOVA with Tukey's post hoc test) regarding the oleic acid content of HSA preparations (see data in Fig. 5 and Fig. S1F).

**Oleic acid content**										
	**HSA1**	**HSA2**	**HSA3**	**HSA4**	**HSA5**	**HSA6**	**HSA7**	**HSA8**	**HSA9**	**HSA10**
**HSA1**	–	<0.01	n.s.	n.s.	n.s.	n.s.	<0.01	<0.01	<0.01	<0.01
**HSA2**	<0.01	–	<0.01	<0.01	<0.01	<0.01	<0.01	<0.01	<0.01	n.s.
**HSA3**	n.s.	<0.01	–	n.s.	n.s.	n.s.	<0.01	<0.01	<0.01	<0.01
**HSA4**	n.s.	<0.01	n.s.	–	n.s.	n.s.	<0.01	<0.01	<0.01	<0.01
**HSA5**	n.s.	<0.01	n.s.	n.s.	–	n.s.	<0.01	n.s.	<0.01	<0.01
**HSA6**	n.s.	<0.01	n.s.	n.s.	n.s.	–	<0.01	<0.01	<0.01	<0.01
**HSA7**	<0.01	<0.01	<0.01	<0.01	<0.01	<0.01	–	n.s.	n.s.	<0.01
**HSA8**	<0.01	<0.01	<0.01	<0.01	n.s.	<0.01	n.s.	–	n.s.	<0.01
**HSA9**	<0.01	<0.01	<0.01	<0.01	<0.01	<0.01	n.s.	n.s.	–	<0.01
**HSA10**	<0.01	n.s.	<0.01	<0.01	<0.01	<0.01	<0.01	<0.01	<0.01	–

n.s. = not significant

Table S7: Analysis of statistical differences (one-way ANOVA with Tukey's post hoc test) regarding the linoleic acid content of HSA preparations (see data in Fig. 5 and Fig. S1G).

**Linoleic acid content**										
	**HSA1**	**HSA2**	**HSA3**	**HSA4**	**HSA5**	**HSA6**	**HSA7**	**HSA8**	**HSA9**	**HSA10**
**HSA1**	–	n.s.	n.s.	n.s.	n.s.	n.s.	n.s.	n.s.	n.s.	<0.01
**HSA2**	n.s.	–	n.s.	<0.01	<0.01	n.s.	<0.01	<0.01	<0.01	<0.01
**HSA3**	n.s.	n.s.	–	n.s.	n.s.	n.s.	n.s.	n.s.	n.s.	<0.01
**HSA4**	n.s.	<0.01	n.s.	–	n.s.	n.s.	n.s.	n.s.	n.s.	<0.01
**HSA5**	n.s.	<0.01	n.s.	n.s.	–	n.s.	n.s.	n.s.	n.s.	<0.01
**HSA6**	n.s.	n.s.	n.s.	n.s.	n.s.	–	n.s.	n.s.	n.s.	<0.01
**HSA7**	n.s.	<0.01	n.s.	n.s.	n.s.	n.s.	–	n.s.	n.s.	<0.01
**HSA8**	n.s.	<0.01	n.s.	n.s.	n.s.	n.s.	n.s.	–	n.s.	<0.01
**HSA9**	n.s.	<0.01	n.s.	n.s.	n.s.	n.s.	n.s.	n.s.	–	<0.01
**HSA10**	<0.01	<0.01	<0.01	<0.01	<0.01	<0.01	<0.01	<0.01	<0.01	–

n.s. = not significant

Table S8: Analysis of statistical differences (one-way ANOVA with Tukey's post hoc test) regarding the total free fatty acid content of HSA preparations (see data in Fig. 5 and Fig. S1H).

**Total free fatty acid content**										
	**HSA1**	**HSA2**	**HSA3**	**HSA4**	**HSA5**	**HSA6**	**HSA7**	**HSA8**	**HSA9**	**HSA10**
**HSA1**	–	n.s.	n.s.	n.s.	n.s.	n.s.	<0.01	<0.01	<0.01	<0.01
**HSA2**	n.s.	–	<0.01	<0.01	<0.01	<0.01	<0.01	<0.01	<0.01	<0.01
**HSA3**	n.s.	<0.01	–	n.s.	n.s.	n.s.	n.s.	n.s.	n.s.	<0.01
**HSA4**	n.s.	<0.01	n.s.	–	n.s.	n.s.	n.s.	n.s.	n.s.	<0.01
**HSA5**	n.s.	<0.01	n.s.	n.s.	–	n.s.	n.s.	n.s.	n.s.	<0.01
**HSA6**	n.s.	<0.01	n.s.	n.s.	n.s.	–	n.s.	n.s.	n.s.	<0.01
**HSA7**	<0.01	<0.01	n.s.	n.s.	n.s.	n.s.	–	n.s.	n.s.	<0.01
**HSA8**	<0.01	<0.01	n.s.	n.s.	n.s.	n.s.	n.s.	–	n.s.	<0.01
**HSA9**	<0.01	<0.01	n.s.	n.s.	n.s.	n.s.	n.s.	n.s.	–	<0.01
**HSA10**	<0.01	<0.01	<0.01	<0.01	<0.01	<0.01	<0.01	<0.01	<0.01	–

n.s. = not significant

Table S9: Analysis of statistical differences (one-way ANOVA with Tukey's post hoc test) regarding the filtered fractions of warfarin (WAR), where samples contained 1 µM WAR + 3.5 µM HSA (see data in Fig. 8A with light orange color). Yellow background marks where we noticed statistically significant differences in the presence of both 3.5 µM (Table S9) and 6 µM (Table S10) HSA concentrations.

**Ultracentrifugation – 1 µM WAR + 3.5 µM HSA**										
	**HSA1**	**HSA2**	**HSA3**	**HSA4**	**HSA5**	**HSA6**	**HSA7**	**HSA8**	**HSA9**	**HSA10**
**HSA1**	–	<0.01	n.s.	n.s.	n.s.	n.s.	<0.01	n.s.	<0.01	<0.01
**HSA2**	<0.01	–	<0.01	<0.01	<0.01	<0.01	<0.01	<0.01	<0.01	n.s.
**HSA3**	n.s.	<0.01	–	n.s.	n.s.	n.s.	n.s.	n.s.	n.s.	<0.01
**HSA4**	n.s.	<0.01	n.s.	–	n.s.	n.s.	n.s.	n.s.	n.s.	<0.01
**HSA5**	n.s.	<0.01	n.s.	n.s.	–	n.s.	<0.01	n.s.	<0.01	<0.01
**HSA6**	n.s.	<0.01	n.s.	n.s.	n.s.	–	n.s.	n.s.	n.s.	<0.01
**HSA7**	<0.01	<0.01	n.s.	n.s.	<0.01	n.s.	–	<0.01	n.s.	<0.01
**HSA8**	n.s.	<0.01	n.s.	n.s.	n.s.	n.s.	<0.01	–	n.s.	<0.01
**HSA9**	<0.01	<0.01	n.s.	n.s.	<0.01	n.s.	n.s.	n.s.	–	<0.01
**HSA10**	<0.01	n.s.	<0.01	<0.01	<0.01	<0.01	<0.01	<0.01	<0.01	–

n.s. = not significant

Table S10: Analysis of statistical differences (one-way ANOVA with Tukey's post hoc test) regarding the filtered fractions of warfarin (WAR), where samples contained 1 µM WAR + 6 µM HSA (see data in Fig. 8A with red color). Yellow background marks where we noticed statistically significant differences in the presence of both 3.5 µM (Table S9) and 6 µM (Table S10) HSA concentrations.

**Ultracentrifugation – 1 µM WAR + 6 µM HSA**										
	**HSA1**	**HSA2**	**HSA3**	**HSA4**	**HSA5**	**HSA6**	**HSA7**	**HSA8**	**HSA9**	**HSA10**
**HSA1**	–	<0.01	n.s.	n.s.	n.s.	n.s.	n.s.	n.s.	<0.01	<0.01
**HSA2**	<0.01	–	<0.01	<0.01	<0.01	<0.01	<0.01	<0.01	<0.01	n.s.
**HSA3**	n.s.	<0.01	–	n.s.	n.s.	n.s.	n.s.	n.s.	n.s.	<0.01
**HSA4**	n.s.	<0.01	n.s.	–	n.s.	n.s.	n.s.	n.s.	n.s.	<0.01
**HSA5**	n.s.	<0.01	n.s.	n.s.	–	n.s.	n.s.	n.s.	<0.01	<0.01
**HSA6**	n.s.	<0.01	n.s.	n.s.	n.s.	–	n.s.	n.s.	<0.01	<0.01
**HSA7**	n.s.	<0.01	n.s.	n.s.	n.s.	n.s.	–	n.s.	n.s.	<0.01
**HSA8**	n.s.	<0.01	n.s.	n.s.	n.s.	n.s.	n.s.	–	n.s.	<0.01
**HSA9**	<0.01	<0.01	n.s.	n.s.	<0.01	<0.01	n.s.	n.s.	–	<0.01
**HSA10**	<0.01	n.s.	<0.01	<0.01	<0.01	<0.01	<0.01	<0.01	<0.01	–

n.s. = not significant

Table S11: Analysis of statistical differences (one-way ANOVA with Tukey's post hoc test) regarding the filtered fractions of naproxen (NAP), where samples contained 1 μM NAP + 1 μM HSA (see data in Fig. 8B with cyan color). Green background marks where we noticed statistically significant differences in the presence of both 1 μM (Table S11) and 1.6 μM (Table S12) HSA concentrations.

**Ultracentrifugation – 1 μM NAP + 1 μM HSA**										
	**HSA1**	**HSA2**	**HSA3**	**HSA4**	**HSA5**	**HSA6**	**HSA7**	**HSA8**	**HSA9**	**HSA10**
**HSA1**	–	<0.01	n.s.	n.s.	<0.01	n.s.	n.s.	n.s.	<0.01	<0.01
**HSA2**	<0.01	–	<0.01	<0.01	n.s.	<0.01	<0.01	<0.01	<0.01	<0.01
**HSA3**	n.s.	<0.01	–	n.s.	n.s.	n.s.	<0.01	n.s.	<0.01	<0.01
**HSA4**	n.s.	<0.01	n.s.	–	n.s.	n.s.	n.s.	n.s.	<0.01	<0.01
**HSA5**	<0.01	n.s.	n.s.	n.s.	–	<0.01	<0.01	n.s.	<0.01	<0.01
**HSA6**	n.s.	<0.01	n.s.	n.s.	<0.01	–	n.s.	n.s.	<0.01	<0.01
**HSA7**	n.s.	<0.01	<0.01	n.s.	<0.01	n.s.	–	<0.01	<0.01	<0.01
**HSA8**	n.s.	<0.01	n.s.	n.s.	n.s.	n.s.	<0.01	–	<0.01	<0.01
**HSA9**	<0.01	<0.01	<0.01	<0.01	<0.01	<0.01	<0.01	<0.01	–	<0.01
**HSA10**	<0.01	<0.01	<0.01	<0.01	<0.01	<0.01	<0.01	<0.01	<0.01	–

n.s. = not significant

Table S12: Analysis of statistical differences (one-way ANOVA with Tukey's post hoc test) regarding the filtered fractions of naproxen (NAP), where samples contained 1 μM NAP + 1.6 μM HSA (see data in Fig. 8B with blue color). Green background marks where we noticed statistically significant differences in the presence of both 1 μM (Table S11) and 1.6 μM (Table S12) HSA concentrations.

**Ultracentrifugation – 1 μM NAP + 1.6 μM HSA**										
	**HSA1**	**HSA2**	**HSA3**	**HSA4**	**HSA5**	**HSA6**	**HSA7**	**HSA8**	**HSA9**	**HSA10**
**HSA1**	–	<0.01	n.s.	n.s.	n.s.	n.s.	<0.01	n.s.	<0.01	<0.01
**HSA2**	<0.01	–	<0.01	<0.01	<0.01	<0.01	<0.01	<0.01	<0.01	<0.01
**HSA3**	n.s.	<0.01	–	n.s.	n.s.	n.s.	<0.01	n.s.	<0.01	<0.01
**HSA4**	n.s.	<0.01	n.s.	–	n.s.	n.s.	<0.01	n.s.	<0.01	<0.01
**HSA5**	n.s.	<0.01	n.s.	n.s.	–	n.s.	<0.01	n.s.	<0.01	<0.01
**HSA6**	n.s.	<0.01	n.s.	n.s.	n.s.	–	<0.01	n.s.	<0.01	<0.01
**HSA7**	<0.01	<0.01	<0.01	<0.01	<0.01	<0.01	–	<0.01	n.s.	<0.01
**HSA8**	n.s.	<0.01	n.s.	n.s.	n.s.	n.s.	<0.01	–	<0.01	<0.01
**HSA9**	<0.01	<0.01	<0.01	<0.01	<0.01	<0.01	n.s.	<0.01	–	<0.01
**HSA10**	<0.01	<0.01	<0.01	<0.01	<0.01	<0.01	<0.01	<0.01	<0.01	–

n.s. = not significant
